# Infection-induced trained immunity: a twist in paradigm of innate host defense and generation of immunological memory

**DOI:** 10.1128/iai.00472-24

**Published:** 2024-12-10

**Authors:** Aayush Bahl, Saurabh Pandey, Roopshali Rakshit, Sashi Kant, Deeksha Tripathi

**Affiliations:** 1 Microbial Pathogenesis and Microbiome Lab, Department of Microbiology, School of Life Sciences, Central University of Rajasthan 206414 , Ajmer, Rajasthan, India; 2 Department of Biochemistry, School of Chemical and Life Sciences, Jamia Hamdard 683434 , New Delhi, Delhi, India; 3 Bacterial Pathogenesis, Boehringer Ingelheim Animal Health USA Inc 519163 , Ames, Iowa, USA; University of California Davis, Davis, California, USA

**Keywords:** trained immunity, infection, adaptive immunity, innate immunity, epigenetic reprogramming, microbial triggers

## Abstract

In contrast to adaptive immunity, which relies on memory T and B cells for long-term pathogen-specific responses, trained immunity involves the enhancement of innate immune responses through cellular reprogramming. Experimental evidence from animal models and human studies supports the concept of trained immunity and its potential therapeutic applications in the development of personalized medicine. However, there remains a huge gap in understanding the mechanisms, identifying specific microbial triggers responsible for the induction of trained immunity. This underscores the importance of investigating the potential role of trained immunity in redefining host defense and highlights future research directions. This minireview will provide a comprehensive summary of the new paradigm of trained immunity or innate memory pathways. It will shed light on infection-induced pathways through non-specific stimulation within macrophages and natural killer cells, which will be further elaborated in multiple disease perspectives caused by infectious agents such as bacteria, fungi, and viruses. The article further elaborates on the biochemical and cellular basis of trained immunity and its impact on disease status during recurrent exposures. The review concludes with a perspective segment discussing potential therapeutic benefits, limitations, and future challenges in this area of study. The review also sheds light upon potential risks involved in the induction of trained immunity.

## INTRODUCTION

The evolution of our understanding of the immune system has been shaped by seminal discoveries and scientific breakthroughs, dating back to the pioneering work of Elie Metchnikoff and Paul Ehrlich in the late 19th and early 20th centuries (1). Metchnikoff’s observations of phagocytic cells engaging and engulfing pathogens laid the groundwork for the concept of innate immunity, highlighting the role of nonspecific cellular defenses in combating infections. Concurrently, Ehrlich’s studies on antibodies and the principle of immune specificity contributed to the elucidation of adaptive immunity, governed by the highly specialized functions of T and B lymphocytes (1).

Infection-induced trained immunity represents a phenomenon where exposure to certain microbial agents results in the priming of the innate immune system to initiate a more vigorous and rapid response upon subsequent encounters with different pathogens (2). Unlike the conventional understanding of immune memory, which predominantly attributes this function to the adaptive arm of the immune system mediated by T and B lymphocytes, trained immunity introduces a paradigm shift by demonstrating that innate immune cells possess memory-like properties (3–5). This heightened responsiveness is characterized by an enhanced capacity of innate immune cells, such as monocytes, macrophages, and natural killer (NK) cells, to recognize and eliminate pathogens, thereby providing an additional layer of defense against infections (2, 6, 7).

Over time, the dichotomy between innate and adaptive immunity became increasingly apparent, with innate immunity serving as the first line of defense against pathogens, while adaptive immunity provided tailored and long-lasting protection through the generation of antigen-specific memory (8, 9). However, the distinction between these two arms of immunity began to blur with the discovery of mechanisms suggesting that innate immune cells possess the capacity for memory and enhanced responsiveness upon re-encounter with pathogens.

This paradigm shift not only expands our understanding of the complexity and plasticity of the immune system but also underscores the dynamic interplay between innate and adaptive immune mechanisms in orchestrating effective host defense. Comprehensive reviews have been published in this area in the past, but most of them were written when the concept was still in its early stages, and others took a piecemeal approach. Other reviews focused solely on a single disease perspective or were limited to immunological aspects. However, the accumulation of new experimental evidence now allows us to organize and consolidate this fragmented knowledge into a more cohesive understanding. This review will examine the mechanisms behind infection-induced trained immunity, assess its impact on host defense and immunopathology, and explore its potential applications in vaccine development and host-directed immunotherapy.

## IMMUNE MEMORY: ADAPTIVE VS INNATE

### Adaptive immunity: specificity and responsiveness

Adaptive immunity is characterized by its specificity and memory, primarily mediated by T and B lymphocytes. Upon encountering antigens, T and B cells undergo clonal expansion and differentiation into effector cells, which eliminate the invading pathogens (10, 11). Importantly, a subset of these antigen-specific lymphocytes persists as memory cells, poised to mount a rapid and robust response upon re-exposure to the same antigen (10, 11).

The development of memory T and B cells is stimulated by antigen presentation and co-stimulatory signals from antigen-presenting cells (APCs), including dendritic cells (11). When activated, T and B cells undergo clonal expansion and differentiation, resulting in a population of effector cells that play a key role in eliminating pathogens. After the resolution of the infection, a subset of these effector cells develops into durable memory cells, ensuring long-term immune surveillance and defense against reinfection (11).

While adaptive immunity confers long-lasting protection against specific pathogens, its effectiveness in mounting a rapid response to newly encountered infections is often limited by the time required for clonal expansion and differentiation of antigen-specific lymphocytes (1). The period of delay between the initial exposure to a pathogen and the activation of an adaptive immune response leaves the host susceptible to the swift multiplication and spread of pathogens, especially in the early stages of infection.

Furthermore, the specificity of adaptive immune memory poses a challenge in providing broad-spectrum protection against a diverse array of pathogens. Memory T and B cells are specific to particular antigens, meaning they can only identify and respond to the antigens specifically (1). As a result, adaptive immune memory may not confer cross-protection against unrelated pathogens or emerging viral variants, necessitating the development of tailored vaccines for each specific pathogen.

### Innate immunity as an additional player in the generation of immunological memory: twist in paradigm of innate host defense

**Fig 1:**
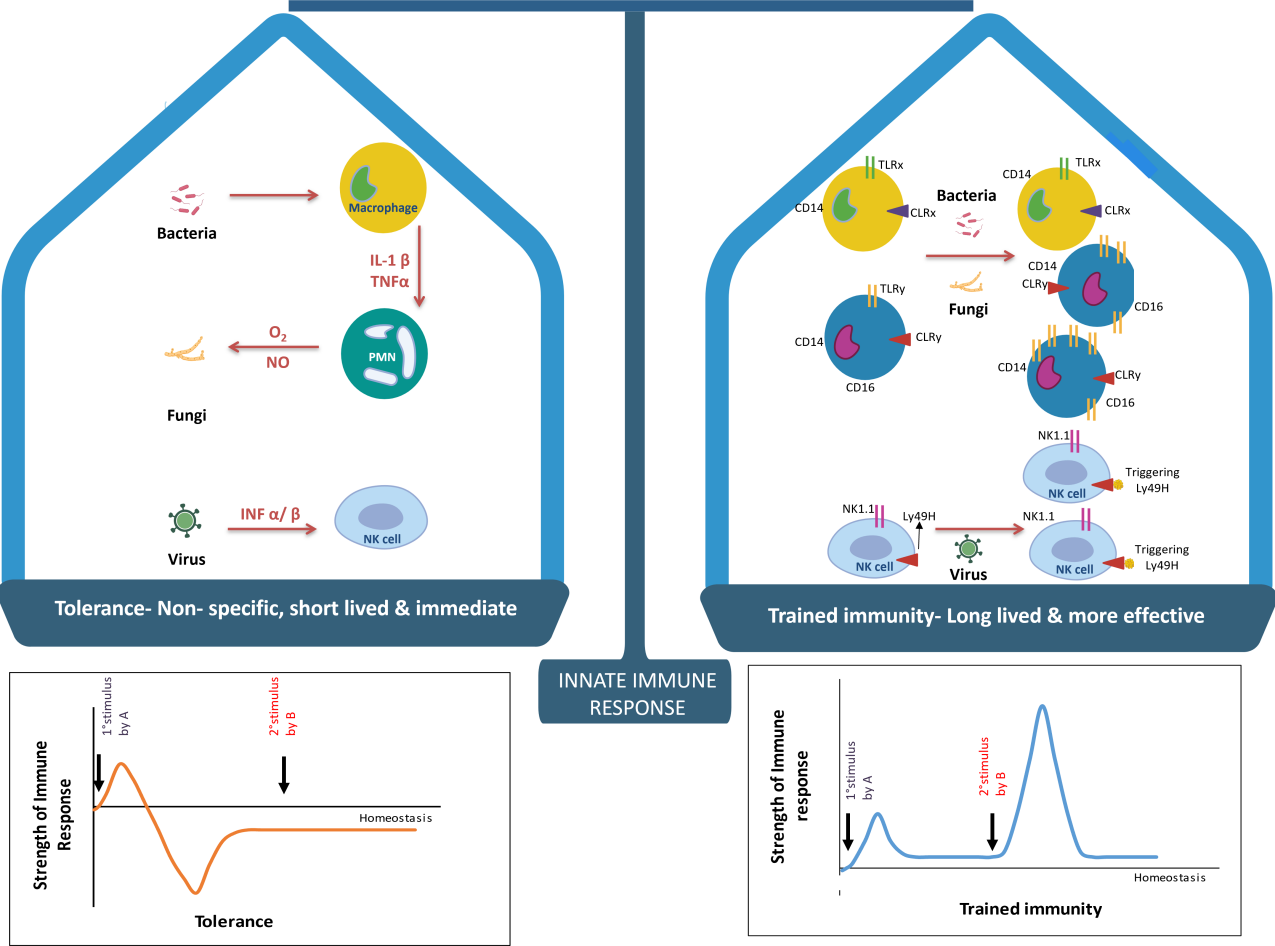
Schematic outline of innate vs trained immunity. The schematic outlines potential changes in the classic dichotomy between innate and trained immunity. In trained immunity, innate host mechanisms can adapt to enhance responses to different pathogens: NK cells may exhibit improved activation of the LY49H receptor (indicated by a star next to the receptor in the figure), while for macrophages, the mechanisms are less well understood. In macrophages, probable mechanisms include variations in monocyte/macrophage subsets (such as CD14 + CD16 and CD14dimCD16+), alterations in pattern recognition receptor (PRR) expression, changes in functional phenotype (like cytokine production), and underlying molecular mechanisms (such as epigenetic modifications and modulation of the miRNA transcriptome). Conversely, innate immune responses are typically rapid and nonspecific, characterized by the release of reactive oxygen and nitrogen species and macrophage activation through interferon-α (IFNα) secretion, and do not involve immunological memory.

Traditionally, innate immunity has been viewed as providing immediate protection against pathogens through mechanisms such as phagocytosis, inflammation, and the activation of antimicrobial peptides. However, emerging evidence suggests that innate immune cells also possess memory-like properties, enabling them to mount a heightened response upon re-exposure to microbial stimuli (2) (Fig. 1). The notion of trained immunity provides a new outlook on immune memory and highlights the importance of innate immune cells in shaping host defense. Trained immunity entails the functional reprogramming of innate immune cells, including monocytes, macrophages, and NK cells (2). Stimulation of immune cells by microbial triggers leads to critical transcriptional changes that initiate immune responses, accompanied by key epigenetic modifications like DNA methylation and histone acetylation. These changes regulate gene expression by reshaping chromatin structure, facilitating the expression or suppression of particular genes, and refining the immune response for future encounter with pathogens (12, 13).

In response to microbial stimuli, the activation of trained immunity is driven by the release of pro-inflammatory cytokines, including interleukin-1β (IL-1β) and tumor necrosis factor-α (TNF-α) (2). These cytokines act on innate immune cells, triggering signaling pathways that result in the activation of transcription factors and the upregulation of genes associated with trained immunity. Both methylation at the cysteine residue and acetylation or methylation at the lysine residue act as transcriptional regulators of the effector genes. Along with that long non-coding RNA (lncRNA) present in the topologically associating domains of the chromatin complex regulate the protein-coding machinery (14). Thus, trained immunity fits into the broader immune landscape, in relation to adaptive immune responses. Immune priming lncRNAs (IPLs) are essential regulators of trained immunity. They are responsible for enhancing histone a modification where three methyl groups are added to the lysine 4 residue of histone H3 (H3K4me3), at promoter regions of genes involved in immune memory. This modification, typically linked with active gene transcription, primes these genes for quicker expression during subsequent immune responses. In the context of trained immunity, where innate immune cells like macrophages and monocytes undergo epigenetic changes, IPLs are key drivers of this process. They help shape the chromatin landscape to support a more efficient immune response upon re-exposure to pathogens (15). H3K4me3 is a histone modification commonly located at gene promoter regions and is linked to active transcription. In monocytes, this mark is found at the promoter sites of genes like TNF, IL6, and IL1B, enhancing their expression during secondary immune challenges. As a result, these genes are primed for quicker activation when immune cells are stimulated again. This process contributes to a more robust immune response during subsequent infections (16–18). Histone modifications induced by IPLs are a key factor contributing to the prolonged immune responses observed in trained immunity. UMLILO (upstream master lncRNA of the inflammatory chemokine locus) is an lncRNA that was found to be very crucial for the deposition of H3K4me3 at the promoter sites of genes such as IL-8, CXCL-1, CXCL-2, and CXCL-3. This regulation plays an essential role in maintaining enhanced immune responsiveness over an extended period (15, 19). Research has demonstrated that UMLILO, when paired with IPL-IL1, is essential for the immunization of neutrophils following Bacillus Calmette-Guerin (BCG) injection. The heightened expression of IPL-IL1 in neutrophils triggers the deposition of H3K4me3 on specific genes. This process ultimately leads to an increase in immune responsiveness. This process underscores the significance of these lncRNAs in promoting an effective immune response (15, 19). The epigenetic modifications involved in the induction of trained immunity elucidate the persistence of trained immunity in effector cells. Other studies also report how the trained immunity, which develops after an initial pathogen exposure, can be inherited by subsequent generations. The study emphasizes the role of epigenetic modifications—such as changes in DNA accessibility and gene expression—in this transgenerational immune enhancement. It reveals that genes related to immune function are more active in these mice compared to controls (20). Trained immunity in microglia, driven by epigenetic reprogramming (e.g., increased H3K4me1 and H3K27ac), exacerbates cerebral β-amyloidosis in Alzheimer’s disease, particularly through HIF-1α and mammalian target of rapamycin (mTOR) pathways. Enhancing histone acetyltransferase activity or inhibiting histone deacetylases (HDACs) could prolong these epigenetic changes and sustain trained immunity. Genes like Tak1 and Hdac1/2, along with cytokine signaling, influence immune memory in microglia, impacting neurodegenerative disease progression (21, 22).

## TRAINED IMMUNITY: CELLULAR PLAYERS AND MECHANISMS

**Fig 2:**
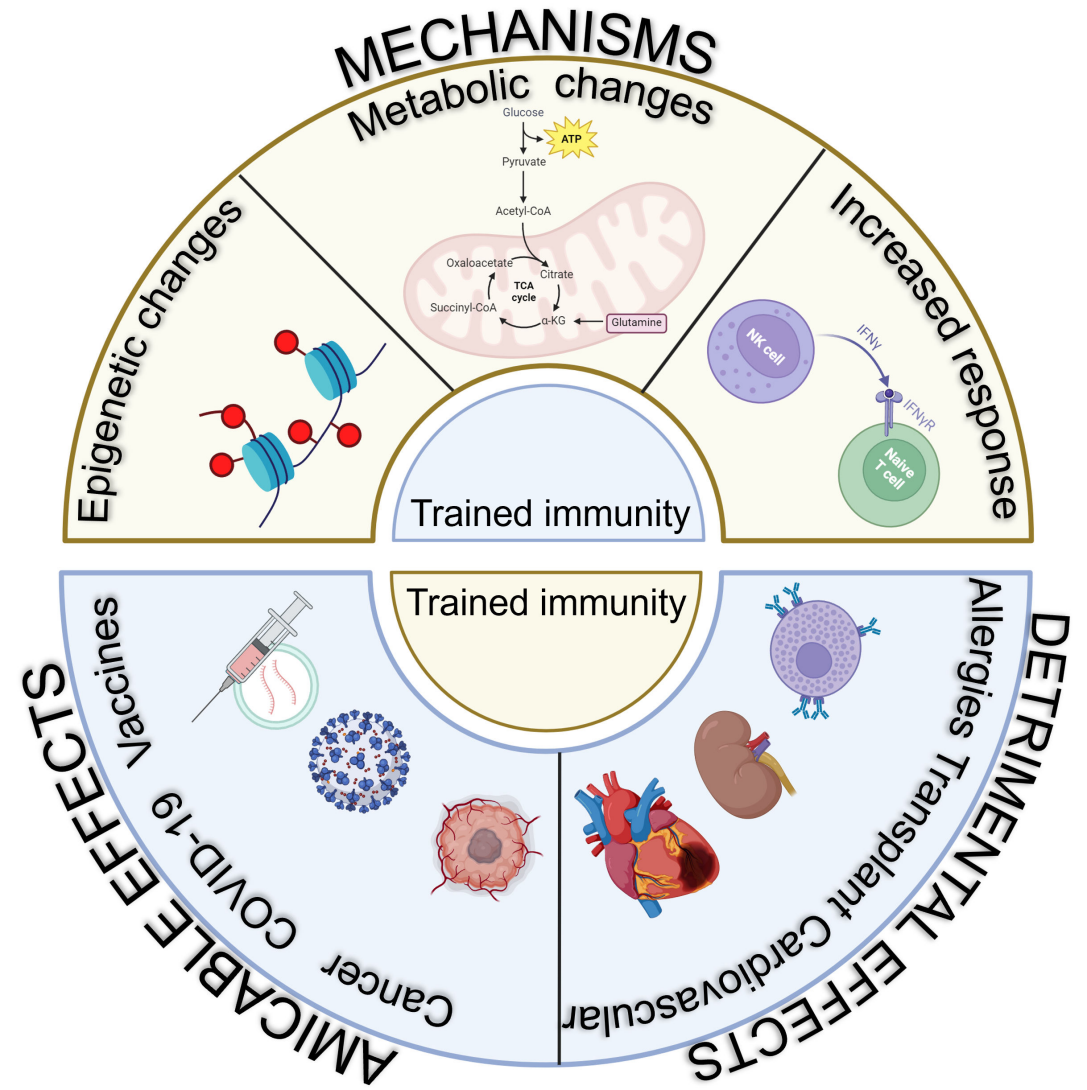
Mechanisms and effects of trained immunity. The mechanisms involved in developing trained immunity are epigenetic changes, metabolic changes, and upregulation of genes expressing cytokines upon encounter with an antigen. This has developed adaptive responses that safeguard the host during subsequent encounter with unrelated pathogens. But, trained immunity is linked to both health and disease, presenting therapeutic potential for either its induction or inhibition, as it can enhance protection against reinfection and optimize vaccination strategies, including for COVID-19. On the contrary, trained immunity can also lead to detrimental effects such as cardiovascular diseases, organ transplant rejection, and severe allergic reactions.

The mechanisms of trained immunity encompass a complex interaction of cellular, signaling, epigenetic, and metabolic pathways that work together to boost the responsiveness of innate immune cells in response to microbial stimuli (Fig. 2).

## NK CELLS, MACROPHAGES, AND OTHER INNATE IMMUNE CELLS

Trained immunity has highlighted NK cells, traditionally known for their role in immune surveillance and cytotoxicity against virus-infected and tumor cells, in a new light. These cells have now emerged as crucial contributors to the mechanisms of trained immunity (23, 24).

Research has demonstrated that trained NK cells display enhanced cytokine production, particularly interferon-gamma (IFN-γ), and enhanced cytotoxic activity against target cells (25, 26). This increased responsiveness is driven by modifications in the expression of activating and inhibitory receptors on the surface of NK cells. Additionally, there are changes in the signaling pathways that regulate NK cell activation and function (27, 28).

APCs release pro-inflammatory cytokines, such as IL-12 and interleukin-18 (IL-18), in response to microbial stimulation by haptens. This cytokine release is crucial for inducing trained immunity in NK cells (29, 30). These cytokines act on NK cells, triggering signaling pathways that lead to the upregulation of genes associated with trained immunity and the acquisition of a memory-like phenotype (31, 32).

Macrophages, serving as expert phagocytes and APCs, are crucial for initiating and regulating immune responses. Trained macrophages exhibit increased expression of pattern recognition receptors (PRRs), such as Toll-like receptors (TLRs) and nucleotide-binding domain (NOD)-like receptors (NLRs), which recognize microbial components and trigger inflammatory signaling cascades (8). The site of infection becomes a focal point for immune activity as pro-inflammatory cytokines are produced, including IL-1β and TNF-α. These cytokines promote the activation and recruitment of immune cells to the affected area (9). There has been evidence of long-lasting effects of trained immunity in experimental studies performed with BCG vaccine. Epigenetic analysis pointed toward the enrichment of H3K4me3 at the promoters for TNF and interleukin-6 (IL-6) in the cytokine-producing macrophages, which were proinflammatory in nature (33–35).

Furthermore, trained macrophages undergo metabolic reprogramming, marked by a shift toward glycolysis and the buildup of particular metabolites like succinate and itaconate (3, 4, 36, 37). These metabolic changes fuel the increased energy demands of trained macrophages and modulate their inflammatory and antimicrobial functions.

In addition to NK cells and macrophages, other innate immune cells, including dendritic cells, neutrophils, and innate lymphoid cells (ILCs), also contribute to trained immunity. Dendritic cells, for example, play a critical role in antigen presentation and the initiation of adaptive immune responses. Trained dendritic cells exhibit increased antigen-presenting capacity and cytokine production, leading to enhanced T-cell activation and effector functions (38, 39). Neutrophils, acting as the initial responders to infection, experience priming and functional enhancement in reaction to microbial stimuli, allowing them to respond more quickly and effectively during later encounters with pathogens. ILCs, including NK cells, type 1 ILCs, and type 3 ILCs, also contribute to trained immunity through their cytokine production and regulatory functions in tissue homeostasis and inflammation (40, 41).

## NONIMMUNE CELLS

Along with immune cells, nonimmune cells, including endothelial cells, epithelial cells, fibroblasts, and vascular smooth muscle cells, are known to play a key role in the induction of trained immunity. Lipopolysaccharide exposure led to the induction of trained immunity in mesenchymal stem cells (42). Epithelial stem cells in the skin are capable of developing immune memory upon microbial infections. Epigenetic changes were involved in the process, and it is called inflammatory memory (43). Exposure to *Pseudomonas aeruginosa* flagellin can elicit trained immunity in bronchial epithelial cells via epigenetic changes (44). Fibroblasts in synovial joints can be activated to show persistent immune response in patients with rheumatoid arthritis (45). Human vascular smooth muscle cells showed enhanced proinflammatory cytokine production upon BCG induction via epigenetic modifications involving mTOR-HIF1α signaling pathway (46).

## INFLAMMATORY SIGNALING

### Role of cytokines (e.g., IL-1β and TNF-α)

In the context of trained immunity, several cytokines play a significant role in mediating the inflammatory response and regulating immune cell functions. IL-1β, TNF-α, IL-6, and IL-18 are crucial mediators of the inflammatory signaling pathways that drive the induction of trained immunity (47, 48).

Upon exposure to microbial stimuli, APCs release pro-inflammatory cytokines that act on innate immune cells, triggering signaling cascades that lead to the activation of transcription factors and the upregulation of genes associated with trained immunity. These cytokines enhance the expression of PRRs, co-stimulatory molecules is enhanced by cytokines, and they also play a role in the formation of memory (49, 50).

### Activation of PRRs

PRRs, such as NLRs, RIG-I-like receptors (RLRs), and TLRs, are essential for identifying microbial pathogens and triggering the innate immune response. In the context of trained immunity, PRRs recognize specific microbial components, such as pathogen-associated molecular patterns (PAMPs) and danger-associated molecular patterns (DAMPs), leading to the activation of inflammatory signaling pathways (51, 52).

Upon activation, PRRs trigger downstream signaling cascades that culminate in the production of pro-inflammatory cytokines, chemokines, and antimicrobial effectors (53, 54). This results in the recruitment and activation of immune cells, such as macrophages, dendritic cells, and neutrophils, to the site of infection, where they mount a coordinated and effective host defense response (55).

## EPIGENETIC REPROGRAMMING

### Histone modifications and DNA methylation

Histone modifications, including acetylation, methylation, phosphorylation, and ubiquitination, have a central role in regulating gene expression and chromatin structure. Changes in histone modifications triggered by exposure to microbial stimuli influence chromatin accessibility. Memory formation and the regulation of genes involved in immune responses are significantly affected by these alterations (5, 56–58).

Lysine-specific demethylase 1 (LSD1) is an enzyme responsible for removing methyl groups from mono- and dimethylated Lys4 of histone H3, a modification tied to gene activation (59, 60). LSD1 enhances macrophage pro-inflammatory specialization by suppressing catalase after TLR4 activation by lipopolysaccharide (LPS). Catalase, which breaks down hydrogen peroxide, limits the expression of pro-inflammatory cytokines and M1-specific markers. Therefore, inhibiting LSD1 could be a therapeutic strategy to suppress hyperactive pro-inflammatory macrophages in certain conditions (61). The second category of histone demethylases consists of a diverse family of proteins characterized by the presence of the Jumonji C (JmjC) domain (62). KDM6A and KDM6B, both members of JmjC methyltransferases, have been reported to enhance the expression of S1pr1, a crucial protein for T cell maturation, along with the transcription factor Klf2. The differentiation of intrathymic T cell precursors into fully mature T cells is a result of the demethylation of H3K27me3 by these enzymes (63). Furthermore, stimulating macrophages with LPS or cytokines activates the NF-κB signaling pathway, leading to increased expression of KDM6B, also a member of JmjC methyltransferases. KDM6B interacts with PcG-target genes to modify their expression by demethylating H3K27me3, thereby enhancing the macrophage’s response to inflammatory stimuli (63). Ten-eleven translocation (TET) proteins, a different category of DNA methyl transferases that function as 5-methylcytosine (5mC) oxidases, have revealed new pathways for reversing DNA methylation (64). TET enzymes convert the methyl group of 5mC into 5-hydroxymethylcytosine and other oxidized methylcytosines. TET proteins play a crucial role in regulating the function of innate immune cells, including macrophages and dendritic cells. In the absence of TET1, LPS-activated mouse macrophages showed increased Il-6 transcription. This increase was associated with changes in CpG methylation at the Il-6 gene locus. Similarly, TET2-deficient mouse peritoneal macrophages exhibited higher basal levels of IL-1β and IL-6 (65, 66). Thus, LSD1, JmjC domain-containing family, and TET family of methyl transferase are integral to the regulation of immunity through its demethylation activity, influencing both innate and adaptive immune responses and maintaining a balance between activation and tolerance in the immune system. Epigenetic modifications, such as H3K4me3 acetylation and demethylation, are critical for trained immunity in tuberculosis, with BCG-trained monocytes exhibiting increased reactive oxygen species (ROS) production, enhanced phagocytosis via CD11 and TLR4 receptors, and NOD2/Rip2-mediated autophagy. Blocking H3K4me3 with 3-methyladenine reverses trained immunity effects. β-glucan induces immune training at the hematopoietic stem and progenitor cell (HSPC) level by enhancing nuclear factor of activated T cells (NFAT)-mediated calcium influx and STAT-1/NF-κB-driven nitric oxide (NO) production, reducing *Mycobacterium tuberculosis* load in macrophages (67).

Studies have indicated that DNA demethylation, particularly in enhancer regions and gene promoters, plays a crucial role in the induction of trained immunity (5, 68). Demethylation of specific genes, such as cytokines and PRRs, promotes their transcriptional activation and the establishment of a memory-like state in innate immune cells. Additionally, DNA methylation inhibitors, such as 5-azacytidine, have been shown to enhance trained immunity and improve host defense against infections (69). The study highlights that trained immunity involves stable changes in histone trimethylation, specifically at H3K4, which leads to cellular activation and enhanced cytokine production by BCG vaccine (6).

## METABOLIC PATHWAYS

### Shift toward glycolysis

A key feature of trained immunity is metabolic reprogramming, which involves a shift in cellular metabolism toward glycolysis. When exposed to microbial stimuli, innate immune cells rapidly adjust their metabolism to satisfy the elevated energy demands associated with their enhanced effector functions (70–73). Glycolysis, the metabolic process in which glucose is broken down to generate energy in the form of ATP, is the primary pathway activated during trained immunity.

Glycolysis typically rises during the activation of immune cells; for instance, activated T cells demonstrate elevated glycolytic rates, while pro-inflammatory macrophages enhance glucose metabolism. Analysis of transcriptional and epigenetic markers (H3K4me3 and H3K27ac) related to β-glucan-induced trained immunity in monocytes showed significant induction of genes involved in the mTOR signaling pathway and various metabolic pathways, particularly glycolysis (74, 75). According to experimental data, activation of the Akt/mTOR/Hif1α pathway mediates the effects of β-glucan-induced training in human monocytes. Inhibiting this pathway at various levels, along with the use of myeloid cell-specific Hif1α knockout mice, abolished the induction of trained immunity, affecting cytokine production and epigenetic changes (74). Glycolytic intermediates, such as pyruvate and lactate, also play important signaling roles in trained immunity, regulating gene expression, cytokine production, and immune cell function.

### Role of metabolites (e.g., succinate and itaconate)

Alongside glycolysis, various metabolites play a role in regulating trained immunity and the functions of immune cells. One such metabolite, succinate, generated in the tricarboxylic acid (TCA) cycle, accumulates in innate immune cells upon exposure to microbial stimuli. This accumulation acts as a signaling molecule that promotes the induction of trained immunity (76, 77).

Succinate acts as a ligand for succinate receptor 1 (SUCNR1), also referred to as GPR91, initiating downstream signaling pathways that activate inflammatory gene expression programs and contribute to the development of a memory-like state in innate immune cells (78–80). Similarly, itaconate, another metabolite of the TCA cycle, has been shown to exert anti-inflammatory effects and modulate immune cell function through its inhibitory effects on succinate dehydrogenase and other enzymes involved in cellular metabolism (81).

The interplay between metabolic pathways and immune signaling in trained immunity highlights the intricate regulatory networks that govern immune cell function and host defense (82). Metabolites generated during cellular metabolism serve not only as energy sources but also as signaling molecules that modulate immune cell activation, differentiation, and memory formation (83, 84). Understanding the metabolic basis of trained immunity may therefore provide new insights into the development of immunomodulatory therapies for infectious diseases and immune-related disorders.

## TRAINED IMMUNITY: COMMONALITIES AND DIFFERENCES AMONG DIVERSE MICROBIAL TRIGGERS

Trained immunity represents a fundamental aspect of the host defense system, enabling innate immune cells to mount a more robust and rapid response upon subsequent encounters with pathogens. Commonalities across different infections contribute to the stimulation and maintenance of trained immunity, even though the specific mechanisms underlying it may vary depending on the nature of the microbial stimulus.

The activation of inflammatory signaling pathways in response to microbial triggers is a key factor that initiates trained immunity. Pro-inflammatory cytokines, such IL-1β, TNF-α, and IL-6, are critical in driving the inflammatory response and facilitating the activation and functional reprogramming of innate immune cells (48, 85–87). As previously noted, PRRs are essential for recognizing microbial pathogens and initiating the innate immune response. These PRRs include TLRs, NLRs, and RLRs. Activation of PRRs by microbial components, such as PAMPs and DAMPs, in turn, results in the activation of downstream signaling pathways that culminate in the increased expression of pro-inflammatory cytokines and the induction of trained immunity (51, 52, 88). While it is established that PAMPs and DAMPS can induce trained immunity, the specific mechanisms by which various pathogens influence the magnitude and quality of this response remain unclear. Research indicates that certain pathogens may elicit stronger or more prolonged training effects than others, suggesting a need for detailed studies on the interactions between specific pathogens and immune cell type. The robust and durable nature of the trained immunity induced by BCG vaccine could be understood by its effects on the bone marrow. Experimental study in mice has revealed that intravenous BCG injection promoted the induction of HSPCs to myelopoiesis, resulting in the production of macrophages that were resistant to *Mycobacterium tuberculosis* infections (89). Similar results were seen in the experiments conducted with human HSPCs with epigenetic changes in the peripheral monocytes upon receiving the injection (90). β-glucan can also induce long-lasting trained immunity via its rector dectin-1. The HSPCs in the bone marrow of mice led to increased myelopoiesis by enhancing the number of LSK (Lin−Sca1+c-Kit+) cells (17). These changes protected the mice against severe *Candida albicans* infection. A generalized increase in cell sensitivity was seen in the study as trained immunity increased the proinflammatory responsiveness of monocytes. This is shown by the heightened production of pro- and anti-inflammatory cytokines, increased generation of ROS, and metabolic activation in response to BCG and oxidized low-density lipoprotein (LDL). Furthermore, adjuvants, which are vital components of vaccines, can significantly enhance the magnitude of the immune response (91, 92). They are included in very weak immunogenic subunit vaccines to induce immune responses, unlike live attenuated vaccines like BCG, which can induce trained immunity by activating PPRs (93). Research has shown that innate immune cells can be trained by various inducers acting like adjuvants like β-glucan, zymosan, and muramyldipeptide providing protection against various secondary infections (17, 69, 94).

Several epidemiological studies have pointed toward the fact that vaccination with live-attenuated microorganisms not only protects against specific pathogens but also reduces all-cause mortality through non-specific effects involving T-cell cross-stimulation and trained immunity (95, 96). Numerous studies have shown that innate immune memory plays a key role in the nonspecific effects of vaccines, particularly with BCG, which induces trained immunity. BCG has been found to protect against unrelated infections in both immunocompromised mice and humans, contributing to reduced childhood mortality and respiratory infections. Animal models show the protection of immunocompromised mice against candidemia; on the other hand, human subjects showed resistance against controlled malaria (97, 98). Recent single-cell RNA sequencing revealed that trained immunity induces heterogeneity in immune cells, with monocytes differentiating into subpopulations such as MCI (expressing chemokines and proinflammatory cytokines) and MC (chemokine-expressing only) (99). MCI cells showed enhanced IL-17 and TNF-α signaling, while MC cells were linked to asthma and type-1 diabetes pathways. Both populations contribute to diseases like bowel disease, ulcerative colitis, and cardiovascular disease, with lymphocyte interactions and pathogen specificity playing critical roles in the training process (99). The heterologous effects of BCG are connected to shifts in metabolism and epigenetic alterations. Heterologous protective effects have been observed in antiviral vaccines, including the oral polio vaccine (OPV), measles-mumps-rubella (MMR), and more recent influenza and COVID-19 vaccines, which can stimulate trained immunity and offer protection against a range of other infections. For instance, OPV not only protects against poliomyelitis but also reduces infant mortality and offers heterologous protection against influenza and respiratory infections. Additionally, influenza vaccines have been shown to cause lasting transcriptional and epigenetic changes in myeloid cells, contributing to enhanced innate immune responses (100, 101). Inducers of trained immunity acting as adjuvants can elicit heterologous protection against secondary infections. Experimental models have shown that β-glucan-induced trained immunity can protect the host from *Leishmania braziliensis*, *Mycobacterium tuberculosis*, *Staphylococcus aureus*, *Leptospira*, and antiviral activity against vesicular stomatitis virus or herpes simplex virus type 1 (102–105).

Trained immunity can be triggered by a diverse array of microbial stimuli, including viruses, bacteria, fungi, and parasites. However, the specific immune responses that arise from different types of infections can vary, influenced by the characteristics of the pathogen and the dynamics of the host-pathogen interaction. Here, we have elucidated the immune responses triggered by viral, bacterial, and fungal infections and their implications for trained immunity.

### Viral infections

Viral infections involve the invasion and replication of viruses inside host cells, triggering antiviral immune responses and the production of type I interferons (IFNs) along with pro-inflammatory cytokines (106, 107). Innate immune cells, including dendritic cells, macrophages, and NK cells, are essential for detecting and responding to viral infections by recognizing viral nucleic acid patterns through PRRs like TLRs and RLRs. When viral PAMPs are recognized, innate immune cells secrete pro-inflammatory cytokines and type I interferons, activating antiviral effector mechanisms and coordinating the adaptive immune response. Trained immunity resulting from viral infections leads to the functional reprogramming of innate immune cells, such as NK cells and monocytes, enabling them to launch a stronger and quicker response upon subsequent exposure to the same or similar viruses (108). Earlier reports have shown that the causative agent of severe hand, foot, and mouth disease, enterovirus 71, possesses a conserved capsid T cell epitope. The epitope is responsible for eliciting a cross-reactive, human leukocyte antigen–DR (HLA-DR)-restricted response of human CD4^+^ T cells to a poliovirus variant of the same epitope (109, 110). Furthermore, studies have revealed that in the case of influenza virus, naïve T cells from healthy donors (not exposed to influenza virus) can recognize unique strain-specific epitopes of H1N1/09. Conversely, memory T cells of the same group identify conserved epitopes of hemagglutinin (111). This is evidence of viral-induced T cell-mediated heterologous immune response.

### Bacterial infection

Bacterial infections elicit a targeted immune response that activates innate immune cells, including neutrophils, macrophages, and dendritic cells, in response to PAMPs like LPS, peptidoglycan, and lipoteichoic acid (112, 113). When bacterial PAMPs are recognized by pattern PRRs, cells responsible for innate immunity generate pro-inflammatory cytokines, chemokines, and antimicrobial effectors that attract and activate other immune cells at the infection site. Trained immunity induced by bacterial infections involves the efficient reprogramming of innate immune cells to enhance their antimicrobial functions and promote the clearance of bacterial pathogens. The long-lasting effects of trained immunity were highlighted in a randomized controlled trial, where one group received the BCG vaccine, while the other received a placebo. Evidence from longitudinal population-based cohort studies also supports the BCG vaccine’s role in promoting enduring protection, mediated by innate immune cells. Experimental studies have also shown that muramyldipeptide acting as an adjuvant can elicit enchased response against *Toxoplasma gondii* (114).

### Fungal infections

Fungal infections trigger immune responses that differ from those caused by viral and bacterial infections, involving the activation of innate immune cells like macrophages, dendritic cells, and neutrophils in response to fungal PAMPs such as β-glucans, mannans, and chitin (55, 115). Pro-inflammatory cytokines, chemokines, and antimicrobial effectors are produced by innate immune cells upon recognition of fungal PAMPs by PRRs. These molecules recruit and activate additional immune cells to the site of infection. Trained immunity induced by fungal infections involves the functional reprogramming of innate immune cells to enhance their antifungal functions and promote the clearance of fungal pathogens. For instance, mice exposed to *Candida albicans* exhibited increased resistance to *Listeria monocytogenes*. This demonstrates the potential of leveraging trained immunity to enhance the body’s defense mechanisms against infections (69). In this study, mice exposed to heat-killed *Candida albicans* displayed significant protection against a deadly *Listeria monocytogenes* infection. These trained mice had a notable reduction in bacteremia, illustrating that prior exposure to one pathogen can offer substantial defense against a different bacterial infection (116–120).

Understanding these commonalities and differences in immune responses to different types of infections is essential for developing strategies to harness trained immunity for vaccine development and therapeutic interventions. Trained immunity is crucial for boosting the immune response to bacterial infections, and *Saccharomyces cerevisiae* exemplifies how initial exposure can prime the immune system for subsequent challenges. In the study, the yeast *Saccharomyces cerevisiae* was found to trigger trained immunity in human monocytes. IL-6 and TNF-α production was elevated in these monocytes when they were later exposed to bacterial or fungal stimuli (115, 121–123).

## TRAINED IMMUNITY: IMPLICATIONS FOR DEVELOPMENT OF VACCINES AND NOVEL THERAPEUTIC INTERVENTION STRATEGIES

The identification of trained immunity holds significant potential for creating innovative therapeutic interventions. By leveraging the innate immune memory triggered by specific microbial stimuli, novel vaccine strategies may be developed to deliver broader and longer-lasting protection against various pathogens. Furthermore, focusing on trained immunity could lead to new approaches for treating inflammatory diseases, autoimmune disorders, and cancer. This could be achieved by modulating the activity of innate immune cells and fostering immune tolerance.

Trained immunity represents a promising avenue to tackle several current challenges in vaccine technology, including vaccine hesitancy, limited efficacy against variants, and the need for protection against unknown pathogens. Live-attenuated vaccines, such as BCG, have long been known to induce nonspecific protection against unrelated infections, suggesting the induction of trained immunity. Recent studies have also demonstrated that certain adjuvants and vaccine formulations can enhance trained immunity and improve vaccine efficacy, highlighting the potential of this approach for optimizing vaccine design and delivery. In HIV vaccine development, various vaccine platforms, including viral vectors and adjuvants, have been recognized for their potential to induce trained immunity. For example, poxviral vectors employed in some HIV vaccine studies can prompt myeloid cells to enter a memory-like state, potentially boosting the vaccine’s overall effectiveness (17, 89, 124). The study of MV130, an inactivated poly-bacterial mucosal vaccine, also demonstrates trained immunity principles by protecting against recurrent respiratory infections and viral diseases. Intranasal MV130 administration reprograms bone marrow progenitor cells and human monocytes, enhancing cytokine production and metabolic shifts associated with trained immunity. This vaccine’s effectiveness, even in Rag1-deficient mice lacking functional lymphocytes, highlights its reliance on innate immunity and its potential to enhance the overall immune response, even in individuals with weakened adaptive immunity (74, 125–127). Vaccines designed for the induction of trained immunity have the potential to provide rapid protection against emerging or unfamiliar pathogens by boosting the innate immune system. For example, in the absence of a specific vaccine for the SARS-CoV-2 at the start of the pandemic, the use of vaccines like BCG with heterologous effects was proposed based on previous studies (33, 128, 129). Experimental data in animal models suggested toward the fact that intravenous BCG-induced human ACE2 transgenic mouse showed efficacy against SARS-CoV-2 (130). However, genetic and environmental factors played a crucial role when the effects of BCG vaccines were studied on human subjects with beneficial and no effect on the disease (131–133). Evidence has also been reported that other vaccines have been assessed for their efficacy against the virus. Different studies have shown that MMR vaccine, OPV, and recombinant adjuvanted zoster vaccine (Shingrix) have shown some positive effects against the virus to some extent, but it needs further investigation to provide concrete evidence (134–136).

Trained immunity offers new opportunities for the development of immunomodulatory therapies for the treatment of infectious diseases and immune-related disorders. By modulating the pathways involved in trained immunity, it may be possible to enhance host defense against a wide range of pathogens, including those that are difficult to target with conventional vaccines (2). Additionally, targeting trained immunity could offer new strategies for the treatment of inflammatory diseases, autoimmune disorders, and cancer by modulating the activity of innate immune cells and promoting immune tolerance (137). For example, therapeutic strategies can adjust macrophage polarization between pro-inflammatory (M1) and anti-inflammatory (M2) states, which is useful in managing conditions like allograft rejection (138, 139). Targeting pathways such as dectin-1 or inhibiting mTOR can enhance immune responses in infections and inflammation (140). N-butyrate, a short-chain fatty acid produced by gut bacteria, has been shown to influence the function of intestinal macrophages, which are key players in innate immunity. By inhibiting HDACs, n-butyrate downregulates proinflammatory mediators such as NO, IL-6, and IL-12, thereby promoting a hyporesponsive state in macrophages toward commensal bacteria (141, 142). This mechanism helps maintain immune tolerance in the gut, preventing unnecessary inflammation against harmless microbes (143). The therapeutic potential of n-butyrate is particularly relevant for conditions like inflammatory bowel disease (IBD), where misregulated immune responses lead to chronic inflammation. Patients with IBD often exhibit reduced levels of short-chain fatty acids (SCFA)-producing bacteria, suggesting that restoring these beneficial microbes or administering SCFA directly could help in managing the disease. Clinical studies have indicated that SCFA enemas can be beneficial for some patients with colitis, highlighting a direct link between therapeutic interventions and innate immune modulation (140). Epigenetic modifications can help balance immune responses in various conditions, while monoclonal antibodies targeting regulators like IL-1β offer promise for managing chronic inflammation (144). Harnessing trained immunity for vaccine development can enhance pathogen clearance and protection against diverse infections. Vaccines can modulate innate immune responses to provide broader protection against viral infections. Experimental studies have demonstrated that BCG can protect against various viral pathogens, including respiratory syncytial virus and influenza A virus. In two separate studies, BCG reduced viral titers of influenza A, leading to decreased inflammation and lung injury (95, 96, 145, 146).

In the context of personalized medicine, trained immunity has the potential to revolutionize the field by providing new insights into the interplay between host genetics, environmental factors, and immune function. By elucidating the factors that contribute to inter-individual variability in trained immunity, it may be possible to develop personalized approaches for harnessing its therapeutic potential. For example, individuals with genetic variants associated with enhanced trained immunity may benefit from immunomodulatory therapies that boost innate immune responses, while those with genetic variants associated with dysregulated trained immunity may benefit from interventions that restore immune homeostasis (5, 127, 147). Environmental exposure to pathogens, pollutants, and lifestyle can also affect the immune system. In recent studies, it has been found that IL-1β, which is involved in the formation of atherosclerotic plaque in cardiovascular patients as a result of excessive trained immunity, can be inhibited by monoclonal antibody approach (148). This points toward the personalized approach for cardiovascular patients in the context of innate immune response. Epigenetic modifications such as histone modifications and DNA methylation are a crucial part of regulating individual immune responses. Personalized medicine approach can take advantage of such research information for patients in requirement of therapies targeting epigenetic modifications. Specifically, in case of cancer patients to inhibit the growth of tumor cells, azacytidine and decitabine can be used as they are DNA methyltransferase inhibitors (149). Personalized vaccination strategies to an individual’s genetic background and environmental factors can result in more efficient immunization methods. In a study, participants received the BCG vaccine at different time intervals in a day. For the induction of the mononuclear cells present in the peripheral blood, *Staphylococcus aureus* and *Mycobacterium tuberculosis* were introduced before vaccination, as well as 2 weeks and 3 months afterward. The results showed that those vaccinated in the morning had a more robust immune response and higher cytokine production in their monocytes compared to those vaccinated in the evening (150). Personalized treatments in patients undergoing transplant might include inhibitors like rapamycin to regulate trained immunity by inhibiting the dectin signaling pathway which plays an important role in stimulation of macrophages (140).

## DETRIMENTAL EFFECTS OF TRAINED IMMUNITY: CHRONIC INFLAMMATION AND AUTOIMMUNITY

Trained immunity provides benefits in combating infections; however, its improper activation can be detrimental in chronic inflammatory conditions such as atherosclerosis, rheumatic diseases, and neurodegenerative disorders. Consequently, this could have a negative impact on the overall health of affected individuals (151).

Trained immunity is the prime candidate for the development of atherosclerosis in mice according to experimental data (152, 153). The condition of myeloid bias emerges in diabetes due to the proliferation of HSPCs in the bone marrow. Macrophages from the bone marrow of diabetic mice exhibit a hyperresponsive trained phenotype. When bone marrow from these diabetic mice is transplanted into normoglycemic mice susceptible to atherosclerosis, it hastens the progression of the disease. Moreover, observational studies in humans reveal that individuals with a familial history of hypercholesterolemia have circulating monocytes that demonstrate traits of trained immunity. This suggests a potential link between trained immunity and atherosclerosis risk in these individuals (154). Patients with familial hypercholesterolemia maintained elevated cytokine production and histone markers after 3 months of statin therapy, suggesting long-term immune memory. Similarly, pheochromocytoma patients exhibited a trained immunity phenotype in monocytes, characterized by enhanced cytokine production and metabolic changes, persisting for up to a month post-tumor removal (155).

Targeting trained immunity to regulate the outcomes is possible at different levels, ranging from the whole-body and organ-system levels to the cellular and subcellular levels. Although targeting mature innate immune cells can provide rapid results, reprogramming HSPCs may offer more substantial therapeutic benefits, as the persistence of trained immunity largely depends on these cells (17, 90). Enhancing or suppressing trained immunity can be achieved by targeting specific receptors at the cellular level. For example, in *Candida albicans*, the β-glucan derived from the cell wall is capable of inducing trained immunity. In contrast, laminarin, a mixture of short-chain β-glucans sourced from seaweeds, acts as a competitive inhibitor by blocking the binding of β-glucan to the dectin-1 receptor. These findings highlight β-glucan’s potential as a therapeutic target for modulating mechanisms induced by polysaccharides (156, 157).

## FUTURE DIRECTIONS

Trained immunity involves complex cellular and molecular mechanisms, including the intricate interplay of inflammatory, metabolic, and epigenetic pathways to reprogram innate immune cells. Understanding how these pathways are activated and regulated, as well as their tissue-specific effects, is key to uncover the full scope of trained immunity. Epigenetic and metabolic reprogramming are central to this process, and ongoing research is aimed at identifying the specific modifications and signaling pathways that contribute to innate immune memory across different cell types and tissues. The complexity of these interactions makes it challenging to fully comprehend the implications of trained immunity in host defense and disease progression.

**Fig 3:**
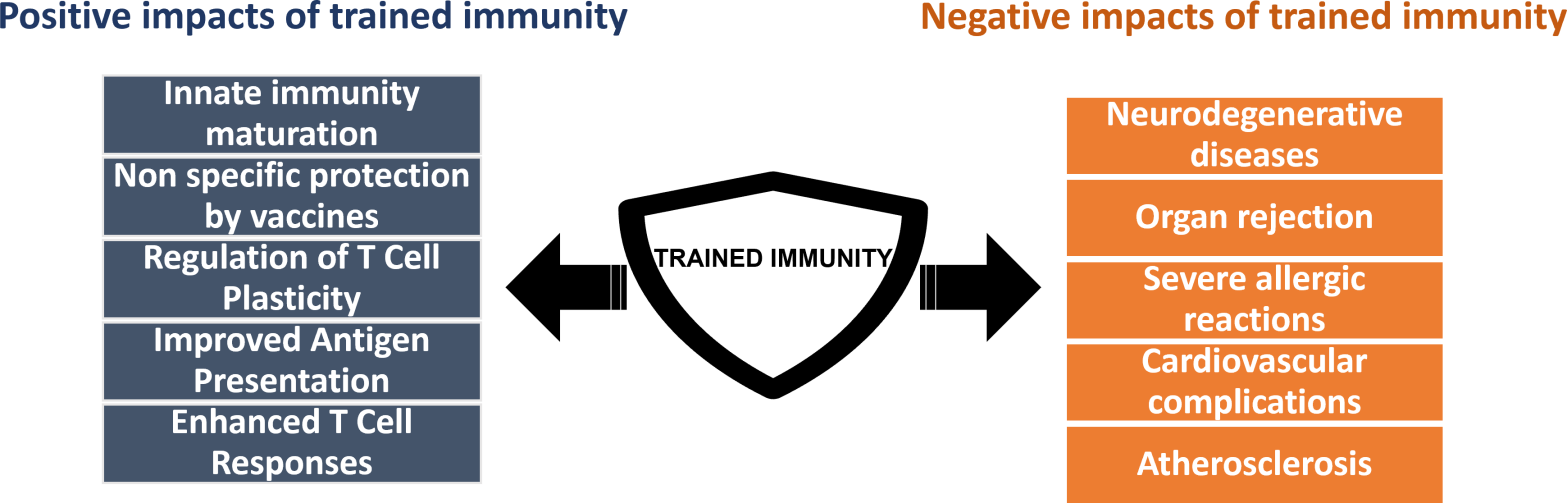
The paradox of trained immunity. Trained immunity has beneficial effects, including enhanced protection against infections like COVID-19, prevention of sepsis, and potential improvements in vaccination strategies. However, its detrimental effects are linked to autoimmune and inflammatory conditions, such as atherosclerosis, neurodegenerative diseases, and allergies, and can also contribute to organ rejection in transplant surgeries.

Excessive or defective trained immunity can have significant consequences for health. In cases of excessive trained immunity, the immune system becomes overactive, potentially leading to harmful inflammatory responses, such as those seen in severe COVID-19 cases or in conditions like hyper-IgD syndrome, where amplified immune signaling causes chronic inflammation. This highlights the importance of balancing immune enhancement with the risk of overactivation (77, 97, 127) (Fig. 3). On the other hand, defective-trained immunity, often seen in diseases like cancer, results in a diminished immune response, increasing susceptibility to infections and possibly promoting tumor growth (97, 151, 158). Epigenetic disruptions in immune cells are thought to underlie these defective responses, though the precise mechanisms remain under investigation.

Trained immunity indeed is a promising avenue for the development of new age vaccine technologies and therapeutics with the onset of patented compounds like BCG and β-glucan-based therapeutic ventures, which include capsules, gels, nanoparticles, and vaccines to control the mechanisms involved in trained immunity (159, 160). However, there is a major drawback in using trained immunity elicitors which is their bioavailability and route of administration. β-glucan being a polysaccharide gets degraded in the digestive system preventing it from stimulating any kind of innate immune response. Different scientific groups have studied the effect of routes of administration of the BCG vaccine. There is a stark difference in the administration of BCG intra-venous primed the hematopoietic cells, whereas a sub-cutaneous administration of the vaccine had so such effects in mice (90, 161). While it is evident that these processes are interconnected and affect one another, it remains uncertain whether the long-lasting effects of trained immunity stem from metabolic events or epigenetic modifications. Thus, understanding the balance between trained immunity’s beneficial and detrimental effects is crucial for developing therapeutic interventions.

## CONCLUSION

Despite the significant progress made in understanding trained immunity, there are still many unanswered questions and unresolved challenges in the field. One key question is the precise mechanisms underlying the induction and maintenance of trained immunity, while epigenetic and metabolic reprogramming have been implicated in mediating the effects of trained immunity (2, 18). The specific signaling pathways and molecular mechanisms involved are still not fully understood.

The long-term effects of trained immunity on health remain uncertain, as dysregulated innate immune activation could lead to chronic inflammation, autoimmune diseases, or other immune disorders. Balancing protective and pathological effects is key for developing immunotherapies. Individual variability in trained immunity, influenced by factors like age, sex, genetics, and environment, complicates its clinical application (162–164). Therefore, further studies are needed to elucidate the factors that contribute to inter-individual variability in trained immunity and to develop personalized approaches for harnessing its therapeutic potential.

Future research will likely focus on further decoding the mechanisms behind trained immunity, identifying specific microbial triggers, and exploring how these findings can translate into clinical applications. With attention to microbial diversity and host-pathogen interactions, researchers aim to develop more effective vaccines and immunotherapies. This growing understanding of trained immunity opens new doors for innovation in disease prevention and treatment, offering exciting prospects for improving global health.

## References

[1] Weaver C, Murphy K. 2024. Janeway’s immunobiology. Google Books. https://books.google.co.in/books?hl=en&lr=&id=GmPLCwAAQBAJ&oi=fnd&pg=PP1&dq=Murphy+and+Weaver,+2016&ots=6crd46y0vj&sig=jEsX6zNQBcKa_ZwTvnKaiVMkVc0#v=onepage&q=Murphy%20and%20Weaver%2C%202016&f=false.

[2] Netea MG, Quintin J, van der Meer JWM. 2011. Trained immunity: a memory for innate host defense. Cell Host Microbe 9:355–361. doi:10.1016/j.chom.2011.04.006 21575907

[3] Sherwood ER, Burelbach KR, McBride MA, Stothers CL, Owen AM, Hernandez A, Patil NK, Williams DL, Bohannon JK. 2022. Innate immune memory and the host response to infection. J Immunol 208:785–792. doi:10.4049/jimmunol.2101058 35115374 PMC8982914

[4] Netea MG. 2013. Training innate immunity: the changing concept of immunological memory in innate host defence. Eur J Clin Invest 43:881–884. doi:10.1111/eci.12132 23869409

[5] Netea MG, Joosten LAB, Latz E, Mills KHG, Natoli G, Stunnenberg HG, O’Neill LAJ, Xavier RJ. 2016. Trained immunity: a program of innate immune memory in health and disease. Science 352:aaf1098. doi:10.1126/science.aaf1098 27102489 PMC5087274

[6] van der Meer JWM, Joosten LAB, Riksen N, Netea MG. 2015. Trained immunity: a smart way to enhance innate immune defence. Mol Immunol 68:40–44. doi:10.1016/j.molimm.2015.06.019 26597205

[7] Gardiner CM, Mills KHG. 2016. The cells that mediate innate immune memory and their functional significance in inflammatory and infectious diseases. Semin Immunol 28:343–350. doi:10.1016/j.smim.2016.03.001 26979658

[8] Takeuchi O, Akira S. 2010. Pattern recognition receptors and inflammation. Cell 140:805–820. doi:10.1016/j.cell.2010.01.022 20303872

[9] Janeway CA, Medzhitov R. 2002. Innate immune recognition. Annu Rev Immunol 20:197–216. doi:10.1146/annurev.immunol.20.083001.084359 11861602

[10] Cooper MD, Alder MN. 2006. The evolution of adaptive immune systems. Cell 124:815–822. doi:10.1016/j.cell.2006.02.001 16497590

[11] Ahmed R, Gray D. 1996. Immunological memory and protective immunity: understanding their relation. Science 272:54–60. doi:10.1126/science.272.5258.54 8600537

[12] Hashimoto H, Vertino PM, Cheng X. 2010. Molecular coupling of DNA methylation and histone methylation. Epigenomics 2:657–669. doi:10.2217/epi.10.44 21339843 PMC3039846

[13] Pongubala JMR, Murre C. 2021. Spatial organization of chromatin: transcriptional control of adaptive immune cell development. Front Immunol 12:633825. doi:10.3389/fimmu.2021.633825 33854505 PMC8039525

[14] Fanucchi S., Shibayama Y, Burd S, Weinberg MS, Mhlanga MM. 2013. Chromosomal contact permits transcription between coregulated genes. Cell 155:606–620. doi:10.1016/j.cell.2013.09.051 24243018

[15] Fanucchi S, Fok ET, Dalla E, Shibayama Y, Börner K, Chang EY, Stoychev S, Imakaev M, Grimm D, Wang KC, Li G, Sung W-K, Mhlanga MM. 2019. Immune genes are primed for robust transcription by proximal long noncoding RNAs located in nuclear compartments. Nat Genet 51:138–150. doi:10.1038/s41588-018-0298-2 30531872

[16] Domínguez-Andrés J, Ferreira AV, Jansen T, Smithers N, Prinjha RK, Furze RC, Netea MG. 2019. Bromodomain inhibitor I-BET151 suppresses immune responses during fungal–immune interaction. Eur J Immunol 49:2044–2050. doi:10.1002/eji.201848081 31206650 PMC6899658

[17] Mitroulis I, Ruppova K, Wang B, Chen L-S, Grzybek M, Grinenko T, Eugster A, Troullinaki M, Palladini A, Kourtzelis I, et al.. 2018. Modulation of myelopoiesis progenitors is an integral component of trained immunity. Cell 172:147–161. doi:10.1016/j.cell.2017.11.034 29328910 PMC5766828

[18] Quintin J, Saeed S, Martens JHA, Giamarellos-Bourboulis EJ, Ifrim DC, Logie C, Jacobs L, Jansen T, Kullberg B-J, Wijmenga C, Joosten LAB, Xavier RJ, van der Meer JWM, Stunnenberg HG, Netea MG. 2012. Candida albicans infection affords protection against reinfection via functional reprogramming of monocytes. Cell Host Microbe 12:223–232. doi:10.1016/j.chom.2012.06.006 22901542 PMC3864037

[19] Moorlag SJCFM, Rodriguez-Rosales YA, Gillard J, Fanucchi S, Theunissen K, Novakovic B, de Bont CM, Negishi Y, Fok ET, Kalafati L, Verginis P, Mourits VP, Koeken VACM, de Bree LCJ, Pruijn GJM, Fenwick C, van Crevel R, Joosten LAB, Joosten I, Koenen H, Mhlanga MM, Diavatopoulos DA, Chavakis T, Netea MG. 2020. BCG vaccination induces long-term functional reprogramming of human neutrophils. Cell Rep 33:108387. doi:10.1016/j.celrep.2020.108387 33207187 PMC7672522

[20] Katzmarski N, Domínguez-Andrés J, Cirovic B, Renieris G, Ciarlo E, Le Roy D, Lepikhov K, Kattler K, Gasparoni G, Händler K, Theis H, Beyer M, van der Meer JWM, Joosten LAB, Walter J, Schultze JL, Roger T, Giamarellos-Bourboulis EJ, Schlitzer A, Netea MG. 2021. Transmission of trained immunity and heterologous resistance to infections across generations. Nat Immunol 22:1382–1390. doi:10.1038/s41590-021-01052-7 34663978

[21] Vinchi F, Ali MS. 2022. Heterogeneous nature of trained innate immune cells in health and disease. Hemasphere 6:e753. doi:10.1097/HS9.0000000000000753 35813100 PMC9259164

[22] Kain BN, Luna P, Hormaechea Agulla D, Maneix L, Morales-Mantilla DE, Le D, Tran B, Florez MA, Toups J, Han H, Jaksik R, Catic A, Shaw C, King KY. 2021. Specificity and heterogeneity of trained immunity in hematopoietic stem and progenitor cells. Blood 138:2149–2149. doi:10.1182/blood-2021-151189

[23] Geary CD, Sun JC. 2017. Memory responses of natural killer cells. Semin Immunol 31:11–19. doi:10.1016/j.smim.2017.08.012 28863960 PMC5724965

[24] Sun JC, Lopez-Verges S, Kim CC, DeRisi JL, Lanier LL. 2011. NK cells and immune “memory” J Immunol 186:1891–1897. doi:10.4049/jimmunol.1003035 21289313 PMC4410097

[25] Romee R, Schneider SE, Leong JW, Chase JM, Keppel CR, Sullivan RP, Cooper MA, Fehniger TA. 2012. Cytokine activation induces human memory-like NK cells. Blood 120:4751–4760. doi:10.1182/blood-2012-04-419283 22983442 PMC3520618

[26] Paolini R, Bernardini G, Molfetta R, Santoni A. 2015. NK cells and interferons. Cytokine Growth Factor Rev 26:113–120. doi:10.1016/j.cytogfr.2014.11.003 25443799

[27] Chen Y, Lu D, Churov A, Fu R. 2020. Research progress on NK cell receptors and their signaling pathways. Mediators Inflamm 2020:1–14. doi:10.1155/2020/6437057 PMC7396059 32774149

[28] Pegram HJ, Andrews DM, Smyth MJ, Darcy PK, Kershaw MH. 2011. Activating and inhibitory receptors of natural killer cells. Immunol Cell Biol 89:216–224. doi:10.1038/icb.2010.78 20567250

[29] O’Leary JG, Goodarzi M, Drayton DL, von Andrian UH. 2006. T cell– and B cell–independent adaptive immunity mediated by natural killer cells. Nat Immunol 7:507–516. doi:10.1038/ni1332 16617337

[30] Peng H, Jiang X, Chen Y, Sojka DK, Wei H, Gao X, Sun R, Yokoyama WM, Tian Z. 2013. Liver-resident NK cells confer adaptive immunity in skin-contact inflammation. J Clin Invest 123:1444–1456. doi:10.1172/JCI66381 23524967 PMC3613925

[31] Terrén I, Orrantia A, Astarloa-Pando G, Amarilla-Irusta A, Zenarruzabeitia O, Borrego F. 2022. Cytokine-induced memory-like NK cells: from the basics to clinical applications. Front Immunol 13:884648. doi:10.3389/FIMMU.2022.884648/BIBTEX 35603208 PMC9114299

[32] Berrien-Elliott MM, Wagner JA, Fehniger TA. 2015. Human cytokine-induced memory-like natural killer cells. J Innate Immun 7:563–571. doi:10.1159/000382019 25924651 PMC4843115

[33] Giamarellos-Bourboulis EJ, Tsilika M, Moorlag S, Antonakos N, Kotsaki A, Domínguez-Andrés J, Kyriazopoulou E, Gkavogianni T, Adami M-E, Damoraki G, Koufargyris P, Karageorgos A, Bolanou A, Koenen H, van Crevel R, Droggiti D-I, Renieris G, Papadopoulos A, Netea MG. 2020. Activate: randomized clinical trial of BCG vaccination against infection in the elderly. Cell 183:315–323. doi:10.1016/j.cell.2020.08.051 32941801 PMC7462457

[34] Abubakar I, Pimpin L, Ariti C, Beynon R, Mangtani P, Sterne JAC, Fine PEM, Smith PG, Lipman M, Elliman D, Watson JM, Drumright LN, Whiting PF, Vynnycky E, Rodrigues LC. 2013. Systematic review and meta-analysis of the current evidence on the duration of protection by bacillus Calmette–Guérin vaccination against tuberculosis. Health Technol Assess 17:1–372, doi:10.3310/hta17370 PMC4781620 24021245

[35] Nguipdop-Djomo P, Heldal E, Rodrigues LC, Abubakar I, Mangtani P. 2016. Duration of BCG protection against tuberculosis and change in effectiveness with time since vaccination in Norway: a retrospective population-based cohort study. Lancet Infect Dis 16:219–226. doi:10.1016/S1473-3099(15)00400-4 26603173

[36] Arts RJW, Joosten LAB, Netea MG. 2018. The potential role of trained immunity in autoimmune and autoinflammatory disorders. Front Immunol 9:339966. doi:10.3389/FIMMU.2018.00298/BIBTEX PMC5826224 29515591

[37] Bahl A, Rakshit R, Pandey S, Tripathi D. 2024. Genome wide screening to discover novel toxin–antitoxin modules in Mycobacterium indicus pranii; perspective on gene acquisition during mycobacterial evolution. Biotechnol Appl Biochem. doi:10.1002/bab.2651 39113212

[38] Palgen J-L, Feraoun Y, Dzangué-Tchoupou G, Joly C, Martinon F, Le Grand R, Beignon A-S. 2021. Optimize prime/boost vaccine strategies: trained immunity as a new player in the game. Front Immunol 12:612747. doi:10.3389/fimmu.2021.612747 33763063 PMC7982481

[39] Guermonprez P, Valladeau J, Zitvogel L, Théry C, Amigorena S. 2002. Antigen presentation and T cell stimulation by dendritic cells. Annu Rev Immunol 20:621–667. doi:10.1146/annurev.immunol.20.100301.064828 11861614

[40] Eberl G, Colonna M, Di Santo JP, McKenzie ANJ. 2015. Innate lymphoid cells: a new paradigm in immunology. Science 348:aaa6566. doi:10.1126/science.aaa6566 25999512 PMC5658207

[41] Kalafati L, Hatzioannou A, Hajishengallis G, Chavakis T. 2023. The role of neutrophils in trained immunity. Immunol Rev 314:142–157. doi:10.1111/imr.13142 36190144 PMC10050090

[42] Liu G-Y, Liu Y, Lu Y, Qin Y-R, Di G-H, Lei Y-H, Liu H-X, Li Y-Q, Wu C, Hu X-W, Duan H-F. 2016. Short-term memory of danger signals or environmental stimuli in mesenchymal stem cells: implications for therapeutic potential. Cell Mol Immunol 13:369–378. doi:10.1038/cmi.2015.11 25942600 PMC4856802

[43] Naik S, Larsen SB, Gomez NC, Alaverdyan K, Sendoel A, Yuan S, Polak L, Kulukian A, Chai S, Fuchs E. 2017. Inflammatory memory sensitizes skin epithelial stem cells to tissue damage. Nature 550:475–480. doi:10.1038/nature24271 29045388 PMC5808576

[44] Bigot J, Guillot L, Guitard J, Ruffin M, Corvol H, Chignard M, Hennequin C, Balloy V. 2020. Respiratory epithelial cells can remember infection: a proof-of-concept study. J Infect Dis 221:1000–1005. doi:10.1093/infdis/jiz569 31678998

[45] Ospelt C, Reedquist KA, Gay S, Tak PP. 2011. Inflammatory memories: is epigenetics the missing link to persistent stromal cell activation in rheumatoid arthritis? Autoimmun Rev 10:519–524. doi:10.1016/j.autrev.2011.04.001 21497675

[46] Schnack L, Sohrabi Y, Lagache SMM, Kahles F, Bruemmer D, Waltenberger J, Findeisen HM. 2019. Mechanisms of trained innate immunity in oxLDL primed human coronary smooth muscle cells. Front Immunol 10:13. doi:10.3389/fimmu.2019.00013 30728822 PMC6351498

[47] Netea MG, Domínguez-Andrés J, Barreiro LB, Chavakis T, Divangahi M, Fuchs E, Joosten LAB, van der Meer JWM, Mhlanga MM, Mulder WJM, Riksen NP, Schlitzer A, Schultze JL, Stabell Benn C, Sun JC, Xavier RJ, Latz E. 2020. Defining trained immunity and its role in health and disease. Nat Rev Immunol 20:375–388. doi:10.1038/s41577-020-0285-6 32132681 PMC7186935

[48] Moorlag SJCFM, Röring RJ, Joosten LAB, Netea MG. 2018. The role of the interleukin-1 family in trained immunity. Immunol Rev 281:28–39. doi:10.1111/IMR.12617 29248003

[49] Wu TYH. 2016. Strategies for designing synthetic immune agonists. Immunology 148:315–325. doi:10.1111/IMM.12622 27213842 PMC4948037

[50] Shekarian T, Valsesia-Wittmann S, Brody J, Michallet MC, Depil S, Caux C, Marabelle A. 2017. Pattern recognition receptors: immune targets to enhance cancer immunotherapy. Ann Oncol 28:1756–1766. doi:10.1093/annonc/mdx179 28444111

[51] Escamilla-Tilch M, Filio-Rodríguez G, García-Rocha R, Mancilla-Herrera I, Mitchison NA, Ruiz-Pacheco JA, Sánchez-García FJ, Sandoval-Borrego D, Vázquez-Sánchez EA. 2013. The interplay between pathogen-associated and danger-associated molecular patterns: an inflammatory code in cancer? Immunol Cell Biol 91:601–610. doi:10.1038/icb.2013.58 24100386

[52] Amarante-Mendes GP, Adjemian S, Branco LM, Zanetti LC, Weinlich R, Bortoluci KR. 2018. Pattern recognition receptors and the host cell death molecular machinery. Front Immunol 9:2379. doi:10.3389/fimmu.2018.02379 30459758 PMC6232773

[53] Olive C. 2012. Pattern recognition receptors: sentinels in innate immunity and targets of new vaccine adjuvants. Expert Rev Vaccines 11:237–256. doi:10.1586/erv.11.189 22309671

[54] Tan X, Sun L, Chen J, Chen ZJ. 2018. Detection of microbial infections through innate immune sensing of nucleic acids. Annu Rev Microbiol 72:447–478. doi:10.1146/annurev-micro-102215-095605 30200854

[55] Ward RA, Vyas JM. 2020. The first line of defense: effector pathways of anti-fungal innate immunity. Curr Opin Microbiol 58:160–165. doi:10.1016/j.mib.2020.10.003 33217703 PMC7746574

[56] Riksen NP, Netea MG. 2021. Immunometabolic control of trained immunity. Mol Aspects Med 77:100897. doi:10.1016/j.mam.2020.100897 32891423 PMC7466946

[57] van der Heijden CDCC, Noz MP, Joosten LAB, Netea MG, Riksen NP, Keating ST. 2018. Epigenetics and trained immunity. Antioxid Redox Signal 29:1023–1040. doi:10.1089/ars.2017.7310 28978221 PMC6121175

[58] de Laval B, Maurizio J, Kandalla PK, Brisou G, Simonnet L, Huber C, Gimenez G, Matcovitch-Natan O, Reinhardt S, David E, Mildner A, Leutz A, Nadel B, Bordi C, Amit I, Sarrazin S, Sieweke MH. 2020. C/EBPβ-dependent epigenetic memory induces trained immunity in hematopoietic stem cells. Cell Stem Cell 26:793. doi:10.1016/j.stem.2020.03.014 32386557

[59] Purohit JS, Chaturvedi MM. 2017. Chromatin and aging, p 205. In Topics in biomedical gerontology

[60] Verma S, Purohit JS, Arora A, Sinha S, Chaturvedi MM. 2021. Liver regeneration: metabolic and epigenetic regulation. Hepatoma Res 2021. doi:10.20517/2394-5079.2020.122

[61] Sobczak M, Strachowska M, Gronkowska K, Karwaciak I, Pułaski Ł, Robaszkiewicz A. 2021. Lsd1 facilitates pro-inflammatory polarization of macrophages by repressing catalase. Cells 10:2465. doi:10.3390/cells10092465 34572113 PMC8469135

[62] Klose RJ, Kallin EM, Zhang Y. 2006. JmjC-domain-containing proteins and histone demethylation. Nat Rev Genet 7:715–727. doi:10.1038/nrg1945 16983801

[63] Manna S, Kim JK, Baugé C, Cam M, Zhao Y, Shetty J, Vacchio MS, Castro E, Tran B, Tessarollo L, Bosselut R. 2015. Histone H3 Lysine 27 demethylases Jmjd3 and Utx are required for T-cell differentiation. Nat Commun 6:8152. doi:10.1038/ncomms9152 26328764 PMC4569738

[64] Pastor WA, Aravind L, Rao A. 2013. TETonic shift: biological roles of TET proteins in DNA demethylation and transcription. Nat Rev Mol Cell Biol 14:341–356. doi:10.1038/nrm3589 23698584 PMC3804139

[65] Cull AH, Snetsinger B, Buckstein R, Wells RA, Rauh MJ. 2017. Tet2 restrains inflammatory gene expression in macrophages. Exp Hematol 55:56–70. doi:10.1016/J.EXPHEM.2017.08.001 28826859

[66] Lian BSX, Kawasaki T, Kano N, Ori D, Ikegawa M, Isotani A, Kawai T. 2022. Regulation of Il6 expression by single CpG methylation in downstream of Il6 transcription initiation site. iScience 25:104118. doi:10.1016/j.isci.2022.104118 35402874 PMC8983349

[67] Zhou J, Lv J, Carlson C, Liu H, Wang H, Xu T, Wu F, Song C, Wang X, Wang T, Qian Z. 2021. Trained immunity contributes to the prevention of Mycobacterium tuberculosis infection, a novel role of autophagy. Emerg Microbes Infect 10:578–588. doi:10.1080/22221751.2021.1899771 33666534 PMC8018485

[68] Domínguez-Andrés J, Fanucchi S, Joosten LAB, Mhlanga MM, Netea MG. 2020. Advances in understanding molecular regulation of innate immune memory. Curr Opin Cell Biol 63:68–75. doi:10.1016/j.ceb.2019.12.006 31991317

[69] Ciarlo E, Heinonen T, Théroude C, Asgari F, Le Roy D, Netea MG, Roger T. 2020. Trained immunity confers broad-spectrum protection against bacterial infections. J Infect Dis 222:1869–1881. doi:10.1093/infdis/jiz692 31889191 PMC7653089

[70] Namgaladze D, Brüne B. 2023. Rapid glycolytic activation accompanying innate immune responses: mechanisms and function. Front Immunol 14:1180488. doi:10.3389/FIMMU.2023.1180488/BIBTEX 37153593 PMC10158531

[71] Stienstra R, Netea-Maier RT, Riksen NP, Joosten LAB, Netea MG. 2017. Specific and complex reprogramming of cellular metabolism in myeloid cells during innate immune responses. Cell Metab 26:142–156. doi:10.1016/j.cmet.2017.06.001 28683282

[72] Lachmandas E, Boutens L, Ratter JM, Hijmans A, Hooiveld GJ, Joosten LAB, Rodenburg RJ, Fransen JAM, Houtkooper RH, van Crevel R, Netea MG, Stienstra R. 2016. Microbial stimulation of different toll-like receptor signalling pathways induces diverse metabolic programmes in human monocytes. Nat Microbiol 2:16246. doi:10.1038/nmicrobiol.2016.246 27991883

[73] Domblides C, Lartigue L, Faustin B. 2018. Metabolic stress in the immune function of T cells, macrophages and dendritic cells. Cells 7:68. doi:10.3390/cells7070068 29966302 PMC6070887

[74] Cheng S-C, Quintin J, Cramer RA, Shepardson KM, Saeed S, Kumar V, Giamarellos-Bourboulis EJ, Martens JHA, Rao NA, Aghajanirefah A, et al.. 2014. mTOR- and HIF-1α–mediated aerobic glycolysis as metabolic basis for trained immunity. Science 345:6204. doi:10.1126/science.1250684 PMC4226238 25258083

[75] Moorlag SJCFM, Khan N, Novakovic B, Kaufmann E, Jansen T, van Crevel R, Divangahi M, Netea MG. 2020. β-glucan induces protective trained immunity against Mycobacterium tuberculosis infection: a key role for IL-1. Cell Rep 31:107634. doi:10.1016/j.celrep.2020.107634 32433977 PMC7242907

[76] Cordes T, Wallace M, Michelucci A, Divakaruni AS, Sapcariu SC, Sousa C, Koseki H, Cabrales P, Murphy AN, Hiller K, Metallo CM. 2016. Immunoresponsive gene 1 and itaconate inhibit succinate dehydrogenase to modulate intracellular succinate levels. J Biol Chem 291:14274–14284. doi:10.1074/jbc.M115.685792 27189937 PMC4933182

[77] Bekkering S, Arts RJW, Novakovic B, Kourtzelis I, van der Heijden CDCC, Li Y, Popa CD, Ter Horst R, van Tuijl J, Netea-Maier RT, van de Veerdonk FL, Chavakis T, Joosten LAB, van der Meer JWM, Stunnenberg H, Riksen NP, Netea MG. 2018. Metabolic induction of trained immunity through the mevalonate pathway. Cell 172:135–146. doi:10.1016/j.cell.2017.11.025 29328908

[78] Li X, Xie L, Qu X, Zhao B, Fu W, Wu B, Wu J. 2020. GPR91, a critical signaling mechanism in modulating pathophysiologic processes in chronic illnesses. FASEB J 34:13091–13105. doi:10.1096/fj.202001037R 32812686

[79] Trauelsen M, Hiron TK, Lin D, Petersen JE, Breton B, Husted AS, Hjorth SA, Inoue A, Frimurer TM, Bouvier M, O’Callaghan CA, Schwartz TW. 2021. Extracellular succinate hyperpolarizes M2 macrophages through SUCNR1/GPR91-mediated Gq signaling. Cell Rep 35:109246. doi:10.1016/j.celrep.2021.109246 34133934

[80] Keiran N, Ceperuelo-Mallafré V, Calvo E, Hernández-Alvarez MI, Ejarque M, Núñez-Roa C, Horrillo D, Maymó-Masip E, Rodríguez MM, Fradera R, de la Rosa JV, Jorba R, Megia A, Zorzano A, Medina-Gómez G, Serena C, Castrillo A, Vendrell J, Fernández-Veledo S. 2019. SUCNR1 controls an anti-inflammatory program in macrophages to regulate the metabolic response to obesity. Nat Immunol 20:581–592. doi:10.1038/s41590-019-0372-7 30962591

[81] Arts RJW, Novakovic B, Ter Horst R, Carvalho A, Bekkering S, Lachmandas E, Rodrigues F, Silvestre R, Cheng S-C, Wang S-Y, et al.. 2016. Glutaminolysis and fumarate accumulation integrate immunometabolic and epigenetic programs in trained immunity. Cell Metab 24:807–819. doi:10.1016/j.cmet.2016.10.008 27866838 PMC5742541

[82] Saeed S, Quintin J, Kerstens HHD, Rao NA, Aghajanirefah A, Matarese F, Cheng S-C, Ratter J, Berentsen K, van der Ent MA, et al.. 2014. Epigenetic programming of monocyte-to-macrophage differentiation and trained innate immunity. Science 345:1251086. doi:10.1126/science.1251086 25258085 PMC4242194

[83] Buck MD, Sowell RT, Kaech SM, Pearce EL. 2017. Metabolic instruction of immunity. Cell 169:570–586. doi:10.1016/j.cell.2017.04.004 28475890 PMC5648021

[84] Patel CH, Powell JD. 2017. Targeting T cell metabolism to regulate T cell activation, differentiation and function in disease. Curr Opin Immunol 46:82–88. doi:10.1016/j.coi.2017.04.006 28521236 PMC5554728

[85] Huang H, Patel DD, Manton KG. 2005. The immune system in aging: roles of cytokines, T cells and NK cells. Front Biosci 10:192–215. doi:10.2741/1521/PDF 15574362

[86] Striz I, Brabcova E, Kolesar L, Sekerkova A. 2014. Cytokine networking of innate immunity cells: a potential target of therapy. Clin Sci 126:593–612. doi:10.1042/CS20130497 24450743

[87] Watkins LR, Nguyen KT, Lee JE, Maier SF. 1999. Dynamic regulation of proinflammatory cytokines. Adv Exp Med Biol 461:153–178. doi:10.1007/978-0-585-37970-8_10 10442173

[88] Pandey S, Kant S, Khawary M, Tripathi D. 2022. Macrophages in microbial pathogenesis: commonalities of defense evasion mechanisms. Infect Immun 90:e0029121. doi:10.1128/IAI.00291-21 34780281 PMC9119111

[89] Kaufmann E, Sanz J, Dunn JL, Khan N, Mendonça LE, Pacis A, Tzelepis F, Pernet E, Dumaine A, Grenier J-C, Mailhot-Léonard F, Ahmed E, Belle J, Besla R, Mazer B, King IL, Nijnik A, Robbins CS, Barreiro LB, Divangahi M. 2018. BCG educates hematopoietic stem cells to generate protective innate immunity against tuberculosis. Cell 172:176–190. doi:10.1016/j.cell.2017.12.031 29328912

[90] Cirovic B, de Bree LCJ, Groh L, Blok BA, Chan J, van der Velden WJFM, Bremmers MEJ, van Crevel R, Händler K, Picelli S, Schulte-Schrepping J, Klee K, Oosting M, Koeken VACM, van Ingen J, Li Y, Benn CS, Schultze JL, Joosten LAB, Curtis N, Netea MG, Schlitzer A. 2020. BCG vaccination in humans elicits trained immunity via the hematopoietic progenitor compartment. Cell Host Microbe 28:322–334. doi:10.1016/j.chom.2020.05.014 32544459 PMC7295478

[91] Coffman RL, Sher A, Seder RA. 2010. Vaccine adjuvants: putting innate immunity to work. Immunity 33:492–503. doi:10.1016/j.immuni.2010.10.002 21029960 PMC3420356

[92] Kawai T, Akira S. 2010. The role of pattern-recognition receptors in innate immunity: update on Toll-like receptors. Nat Immunol 11:373–384. doi:10.1038/ni.1863 20404851

[93] Pulendran B, S Arunachalam P, O’Hagan DT. 2021. Emerging concepts in the science of vaccine adjuvants. Nat Rev Drug Discov 20:454–475. doi:10.1038/s41573-021-00163-y 33824489 PMC8023785

[94] Lee A, Wimmers F, Pulendran B. 2022. Epigenetic adjuvants: durable reprogramming of the innate immune system with adjuvants. Curr Opin Immunol 77:102189. doi:10.1016/j.coi.2022.102189 35588691 PMC9924100

[95] Higgins JPT, Soares-Weiser K, López-López JA, Kakourou A, Chaplin K, Christensen H, Martin NK, Sterne JAC, Reingold AL. 2016. Association of BCG, DTP, and measles containing vaccines with childhood mortality: systematic review. BMJ 355:i5170. doi:10.1136/bmj.i5170 27737834 PMC5063034

[96] Benn CS, Netea MG, Selin LK, Aaby P. 2013. A small jab – a big effect: nonspecific immunomodulation by vaccines. Trends Immunol 34:431–439. doi:10.1016/j.it.2013.04.004 23680130

[97] Kleinnijenhuis J, Quintin J, Preijers F, Joosten LAB, Ifrim DC, Saeed S, Jacobs C, van Loenhout J, de Jong D, Stunnenberg HG, Xavier RJ, van der Meer JWM, van Crevel R, Netea MG. 2012. Bacille Calmette-Guérin induces NOD2-dependent nonspecific protection from reinfection via epigenetic reprogramming of monocytes. Proc Natl Acad Sci U S A 109:17537–17542. doi:10.1073/pnas.1202870109 22988082 PMC3491454

[98] Walk J, de Bree LCJ, Graumans W, Stoter R, van Gemert G-J, van de Vegte-Bolmer M, Teelen K, Hermsen CC, Arts RJW, Behet MC, Keramati F, Moorlag SJCFM, Yang ASP, van Crevel R, Aaby P, de Mast Q, van der Ven AJAM, Stabell Benn C, Netea MG, Sauerwein RW. 2019. Outcomes of controlled human malaria infection after BCG vaccination. Nat Commun 10:874. doi:10.1038/s41467-019-08659-3 30787276 PMC6382772

[99] Wendeln A-C, Degenhardt K, Kaurani L, Gertig M, Ulas T, Jain G, Wagner J, Häsler LM, Wild K, Skodras A, Blank T, Staszewski O, Datta M, Centeno TP, Capece V, Islam MR, Kerimoglu C, Staufenbiel M, Schultze JL, Beyer M, Prinz M, Jucker M, Fischer A, Neher JJ. 2018. Innate immune memory in the brain shapes neurological disease hallmarks. Nature 556:332–338. doi:10.1038/s41586-018-0023-4 29643512 PMC6038912

[100] Nielsen S, Sujan HM, Benn CS, Aaby P, Hanifi SMA. 2021. Oral polio vaccine campaigns may reduce the risk of death from respiratory infections. Vaccines (Basel) 9:1133. doi:10.3390/vaccines9101133 34696241 PMC8537441

[101] Aaby P, Benn CS. 2019. Developing the concept of beneficial non-specific effect of live vaccines with epidemiological studies. Clin Microbiol Infect 25:1459–1467. doi:10.1016/j.cmi.2019.08.011 31449870

[102] Wang J, Jin Z, Zhang W, Xie X, Song N, Lv T, Wu D, Cao Y. 2019. The preventable efficacy of β-glucan against leptospirosis. PLoS Negl Trop Dis 13:e0007789. doi:10.1371/journal.pntd.0007789 31675378 PMC6860453

[103] dos Santos JC, Barroso de Figueiredo AM, Teodoro Silva MV, Cirovic B, de Bree LCJ, Damen MSMA, Moorlag SJCFM, Gomes RS, Helsen MM, Oosting M, Keating ST, Schlitzer A, Netea MG, Ribeiro-Dias F, Joosten LAB. 2019. β-glucan-induced trained immunity protects against Leishmania braziliensis infection: a crucial role for IL-32. Cell Rep 28:2659–2672. doi:10.1016/j.celrep.2019.08.004 31484076

[104] Di Luzio NR, Williams DL. 1978. Protective effect of glucan against systemic Staphylococcus aureus septicemia in normal and leukemic mice. Infect Immun 20:804–810. doi:10.1128/iai.20.3.804-810.1978 352959 PMC421929

[105] Wang G, Li Z, Tian M, Cui X, Ma J, Liu S, Ye C, Yuan L, Qudus MS, Afaq U, Wu K, Liu X, Zhu C. 2023. β-glucan induces training immunity to promote antiviral activity by activating TBK1. Viruses 15:1204. doi:10.3390/v15051204 37243289 PMC10221698

[106] Dalskov L, Gad HH, Hartmann R. 2023. Viral recognition and the antiviral interferon response. EMBO J 42:e112907. doi:10.15252/embj.2022112907 37367474 PMC10350828

[107] Lee AJ, Ashkar AA. 2018. The dual nature of type I and type II interferons. Front Immunol 9:403701. doi:10.3389/FIMMU.2018.02061/BIBTEX PMC6141705 30254639

[108] Taks EJM, Moorlag SJCFM, Netea MG, van der Meer JWM. 2022. Shifting the immune memory paradigm: trained immunity in viral infections. Annu Rev Virol 9:469–489. doi:10.1146/annurev-virology-091919-072546 35676081

[109] Wei R, Yang C, Zeng M, Terry F, Zhu K, Yang C, Altmeyer R, Martin W, De Groot AS, Leng Q. 2012. A dominant EV71-specific CD4^+^ T cell epitope is highly conserved among human enteroviruses. PLoS One 7:e51957. doi:10.1371/journal.pone.0051957 23251663 PMC3522610

[110] Gern JE, Dick EC, Kelly EAB, Vrtis R, Klein B. 1997. Rhinovirus-specific T cells recognize both shared and serotype-restricted viral epitopes. J Infect Dis 175:1108–1114. doi:10.1086/516449 9129073

[111] Yang J, James E, Gates TJ, DeLong JH, LaFond RE, Malhotra U, Kwok WW. 2013. CD4^+^ T cells recognize unique and conserved 2009 H1N1 influenza hemagglutinin epitopes after natural infection and vaccination. Int Immunol 25:447–457. doi:10.1093/intimm/dxt005 23524391 PMC3720064

[112] Chandler CE, Ernst RK. 2017. Bacterial lipids: powerful modifiers of the innate immune response. F1000Res 6:1334. doi:10.12688/f1000research.11388.1 PMC5553087 28868130

[113] Zanoni I, Granucci F. 2010. Differences in lipopolysaccharide-induced signaling between conventional dendritic cells and macrophages. Immunobiology 215:709–712. doi:10.1016/j.imbio.2010.05.026 20579765

[114] Krahenbuhl JL, Sharma SD, Ferraresi RW, Remington JS. 1981. Effects of muramyl dipeptide treatment on resistance to infection with Toxoplasma gondii in mice. Infect Immun 31:716–722. doi:10.1128/iai.31.2.716-722.1981 7216470 PMC351369

[115] Conrath U. 2011. Molecular aspects of defence priming. Trends Plant Sci 16:524–531. doi:10.1016/j.tplants.2011.06.004 21782492

[116] van der Poll T, van de Veerdonk FL, Scicluna BP, Netea MG. 2017. The immunopathology of sepsis and potential therapeutic targets. Nat Rev Immunol 17:407–420. doi:10.1038/nri.2017.36 28436424

[117] Singer M, Deutschman CS, Seymour CW, Shankar-Hari M, Annane D, Bauer M, Bellomo R, Bernard GR, Chiche J-D, Coopersmith CM, Hotchkiss RS, Levy MM, Marshall JC, Martin GS, Opal SM, Rubenfeld GD, van der Poll T, Vincent J-L, Angus DC. 2016. The third international consensus definitions for sepsis and septic shock (Sepsis-3). JAMA 315:801. doi:10.1001/jama.2016.0287 26903338 PMC4968574

[118] Savva A, Roger T. 2013. Targeting toll-like receptors: promising therapeutic strategies for the management of sepsis-associated pathology and infectious diseases. Front Immunol 4:387. doi:10.3389/fimmu.2013.00387 24302927 PMC3831162

[119] Ciarlo E, Savva A, Roger T. 2013. Epigenetics in sepsis: targeting histone deacetylases. Int J Antimicrob Agents 42:S8–S12. doi:10.1016/j.ijantimicag.2013.04.004 23664675

[120] Venet F, Monneret G. 2018. Advances in the understanding and treatment of sepsis-induced immunosuppression. Nat Rev Nephrol 14:121–137. doi:10.1038/nrneph.2017.165 29225343

[121] Rizzetto L, Ifrim DC, Moretti S, Tocci N, Cheng S-C, Quintin J, Renga G, Oikonomou V, De Filippo C, Weil T, Blok BA, Lenucci MS, Santos MAS, Romani L, Netea MG, Cavalieri D. 2016. Fungal chitin induces trained immunity in human monocytes during cross-talk of the host with Saccharomyces cerevisiae. J Biol Chem 291:7961–7972. doi:10.1074/jbc.M115.699645 26887946 PMC4825003

[122] Quintin J, Cheng S-C, van der Meer JW, Netea MG. 2014. Innate immune memory: towards a better understanding of host defense mechanisms. Curr Opin Immunol 29:1–7. doi:10.1016/j.coi.2014.02.006 24637148

[123] Kurtz J. 2005. Specific memory within innate immune systems. Trends Immunol 26:186–192. doi:10.1016/j.it.2005.02.001 15797508

[124] Kleinnijenhuis J, Quintin J, Preijers F, Benn CS, Joosten LAB, Jacobs C, van Loenhout J, Xavier RJ, Aaby P, van der Meer JWM, van Crevel R, Netea MG. 2014. Long-lasting effects of BCG vaccination on both heterologous Th1/Th17 responses and innate trained immunity. J Innate Immun 6:152–158. doi:10.1159/000355628 24192057 PMC3944069

[125] Brandi P, Conejero L, Cueto FJ, Martínez-Cano S, Dunphy G, Gómez MJ, Relaño C, Saz-Leal P, Enamorado M, Quintas A, Dopazo A, Amores-Iniesta J, Del Fresno C, Nistal-Villán E, Ardavín C, Nieto A, Casanovas M, Subiza JL, Sancho D. 2022. Trained immunity induction by the inactivated mucosal vaccine MV130 protects against experimental viral respiratory infections. Cell Rep 38:110184. doi:10.1016/j.celrep.2021.110184 34986349 PMC8755442

[126] Geller A, Yan J. 2020. Could the induction of trained immunity by β-glucan serve as a defense against COVID-19? Front Immunol 11:557208. doi:10.3389/FIMMU.2020.01782/BIBTEX PMC7372085 32760409

[127] Arts RJW, Moorlag SJCFM, Novakovic B, Li Y, Wang S-Y, Oosting M, Kumar V, Xavier RJ, Wijmenga C, Joosten LAB, Reusken CBEM, Benn CS, Aaby P, Koopmans MP, Stunnenberg HG, van Crevel R, Netea MG. 2018. BCG vaccination protects against experimental viral infection in humans through the induction of cytokines associated with trained immunity. Cell Host Microbe 23:89–100. doi:10.1016/j.chom.2017.12.010 29324233

[128] Netea MG, Giamarellos-Bourboulis EJ, Domínguez-Andrés J, Curtis N, van Crevel R, van de Veerdonk FL, Bonten M. 2020. Trained immunity: a tool for reducing susceptibility to and the severity of SARS-CoV-2 infection. Cell 181:969–977. doi:10.1016/j.cell.2020.04.042 32437659 PMC7196902

[129] Chumakov K, Benn CS, Aaby P, Kottilil S, Gallo R. 2020. Can existing live vaccines prevent COVID-19? Science 368:1187–1188. doi:10.1126/science.abc4262 32527819

[130] Hilligan KL, Namasivayam S, Clancy CS, O’Mard D, Oland SD, Robertson SJ, Baker PJ, Castro E, Garza NL, Lafont BAP, Johnson R, Ronchese F, Mayer-Barber KD, Best SM, Sher A. 2022. Intravenous administration of BCG protects mice against lethal SARS-CoV-2 challenge. J Exp Med 219:2. doi:10.1084/jem.20211862 PMC8669500 34889942

[131] Tsilika M, Taks E, Dolianitis K, Kotsaki A, Leventogiannis K, Damoulari C, Kostoula M, Paneta M, Adamis G, Papanikolaou I, et al.. 2022. ACTIVATE-2: a double-blind randomized trial of BCG vaccination against COVID-19 in individuals at risk. Front Immunol 13:873067. doi:10.3389/fimmu.2022.873067 35865520 PMC9294453

[132] Dos Anjos LRB, da Costa AC, Cardoso A da R, Guimarães RA, Rodrigues RL, Ribeiro KM, Borges KCM, Carvalho A de O, Dias CIS, Rezende A de O, Souza C de C, Ferreira RRM, Saraiva G, Barbosa L de S, Vieira T da S, Conte MB, Rabahi MF, Kipnis A, Junqueira-Kipnis AP. 2022. Efficacy and safety of BCG revaccination with M. bovis BCG Moscow to prevent COVID-19 infection in health care workers: a randomized phase II clinical trial. Front Immunol 13:841868. doi:10.3389/fimmu.2022.841868 35392074 PMC8981724

[133] Moorlag SJCFM, Taks E, Ten Doesschate T, van der Vaart TW, Janssen AB, Müller L, Ostermann P, Dijkstra H, Lemmers H, Simonetti E, Mazur M, Schaal H, Ter Heine R, van de Veerdonk FL, Bleeker-Rovers CP, van Crevel R, Ten Oever J, de Jonge MI, Bonten MJ, van Werkhoven CH, Netea MG. 2022. Efficacy of BCG vaccination against respiratory tract infections in older adults during the coronavirus disease 2019 pandemic. Clin Infect Dis 75:e938–e946. doi:10.1093/cid/ciac182 35247264 PMC8903481

[134] Fedrizzi EN, Girondi JBR, Sakae TM, Steffens SM, Silvestrin AN de S, Claro GS, Iskenderian HA, Hillmann B, Gervasi L, Trapani A Junior, et al.. 2021. Efficacy of the measles-mumps-rubella (MMR) vaccine in the reducing the severity of COVID-19: an interim analysis of a randomised controlled clinical trial. medRxiv. doi:10.1101/2021.09.14.21263598

[135] Yagovkina NV, Zheleznov LM, Subbotina KA, Tsaan AA, Kozlovskaya LI, Gordeychuk IV, Korduban AK, Ivin YY, Kovpak AA, Piniaeva AN, Shishova AA, Shustova EY, Khapchaev YK, Karganova GG, Siniugina AA, Pomaskina TV, Erovichenkov AA, Chumakov K, Ishmukhametov AA. 2022. Vaccination with oral polio vaccine reduces COVID-19 incidence. Front Immunol 13:907341. doi:10.3389/fimmu.2022.907341 35711442 PMC9196174

[136] Li C, Lee A, Grigoryan L, Arunachalam PS, Scott MKD, Trisal M, Wimmers F, Sanyal M, Weidenbacher PA, Feng Y, Adamska JZ, Valore E, Wang Y, Verma R, Reis N, Dunham D, O’Hara R, Park H, Luo W, Gitlin AD, Kim P, Khatri P, Nadeau KC, Pulendran B. 2022. Mechanisms of innate and adaptive immunity to the Pfizer-BioNTech BNT162b2 vaccine. Nat Immunol 23:543–555. doi:10.1038/s41590-022-01163-9 35288714 PMC8989677

[137] Chavakis T, Mitroulis I, Hajishengallis G. 2019. Hematopoietic progenitor cells as integrative hubs for adaptation to and fine-tuning of inflammation. Nat Immunol 20:802–811. doi:10.1038/s41590-019-0402-5 31213716 PMC6709414

[138] Ochando J, Fayad ZA, Madsen JC, Netea MG, Mulder WJM. 2020. Trained immunity in organ transplantation. Am J Transplant 20:10–18. doi:10.1111/ajt.15620 31561273 PMC6940521

[139] Braza MS, van Leent MMT, Lameijer M, Sanchez-Gaytan BL, Arts RJW, Pérez-Medina C, Conde P, Garcia MR, Gonzalez-Perez M, Brahmachary M, et al.. 2018. Inhibiting inflammation with myeloid cell-specific nanobiologics promotes organ transplant acceptance. Immunity 49:819–828. doi:10.1016/j.immuni.2018.09.008 30413362 PMC6251711

[140] Chang PV, Hao L, Offermanns S, Medzhitov R. 2014. The microbial metabolite butyrate regulates intestinal macrophage function via histone deacetylase inhibition. Proc Natl Acad Sci U S A 111:2247–2252. doi:10.1073/pnas.1322269111 24390544 PMC3926023

[141] Atarashi K, Nishimura J, Shima T, Umesaki Y, Yamamoto M, Onoue M, Yagita H, Ishii N, Evans R, Honda K, Takeda K. 2008. ATP drives lamina propria T_H_17 cell differentiation. Nature 455:808–812. doi:10.1038/nature07240 18716618

[142] Bandara HMHN, Lam OLT, Jin LJ, Samaranayake L. 2012. Microbial chemical signaling: a current perspective. Crit Rev Microbiol 38:217–249. doi:10.3109/1040841X.2011.652065 22300377

[143] Kanauchi O, Andoh A, Iwanaga T, Fujiyama Y, Mitsuyama K, Toyonaga A, Bamba T. 1999. Germinated barley foodstuffs attenuate colonic mucosal damage and mucosal nuclear factor kappa B activity in a spontaneous colitis model. J Gastroenterol Hepatol 14:1173–1179. doi:10.1046/j.1440-1746.1999.02025.x 10634153

[144] Brown GD, Herre J, Williams DL, Willment JA, Marshall ASJ, Gordon S. 2003. Dectin-1 mediates the biological effects of β-glucans. J Exp Med 197:1119–1124. doi:10.1084/jem.20021890 12719478 PMC2193964

[145] Mukherjee S, Subramaniam R, Chen H, Smith A, Keshava S, Shams H. 2017. Boosting efferocytosis in alveolar space using BCG vaccine to protect host against influenza pneumonia. PLoS One 12:e0180143. doi:10.1371/journal.pone.0180143 28686604 PMC5501455

[146] Moorlag SJCFM, Arts RJW, van Crevel R, Netea MG. 2019. Non-specific effects of BCG vaccine on viral infections. Clin Microbiol Infect 25:1473–1478. doi:10.1016/j.cmi.2019.04.020 31055165

[147] Nemes E, Geldenhuys H, Rozot V, Rutkowski KT, Ratangee F, Bilek N, Mabwe S, Makhethe L, Erasmus M, Toefy A, et al.. 2018. Prevention of M. tuberculosis infection with H4:IC31 vaccine or BCG revaccination. N Engl J Med 379:138–149. doi:10.1056/NEJMoa1714021 29996082 PMC5937161

[148] Ridker PM, Everett BM, Thuren T, MacFadyen JG, Chang WH, Ballantyne C, Fonseca F, Nicolau J, Koenig W, Anker SD, et al.. 2017. Antiinflammatory therapy with canakinumab for atherosclerotic disease. N Engl J Med 377:1119–1131. doi:10.1056/NEJMoa1707914 28845751

[149] Tough DF, Lewis HD, Rioja I, Lindon MJ, Prinjha RK. 2014. Epigenetic pathway targets for the treatment of disease: accelerating progress in the development of pharmacological tools: IUPHAR Review 11. Br J Pharmacol 171:4981–5010. doi:10.1111/bph.12848 25060293 PMC4253452

[150] de Bree LCJ, Mourits VP, Koeken VACM, Moorlag SJCFM, Janssen R, Folkman L, Barreca D, Krausgruber T, Fife-Gernedl V, Novakovic B, Arts RJW, Dijkstra H, Lemmers H, Bock C, Joosten LAB, van Crevel R, Benn CS, Netea MG. 2020. Circadian rhythm influences induction of trained immunity by BCG vaccination. J Clin Invest 130:5603–5617. doi:10.1172/JCI133934 32692732 PMC7641012

[151] Bekkering S, Quintin J, Joosten LAB, van der Meer JWM, Netea MG, Riksen NP. 2014. Oxidized low-density lipoprotein induces long-term proinflammatory cytokine production and foam cell formation via epigenetic reprogramming of monocytes. Arterioscler Thromb Vasc Biol 34:1731–1738. doi:10.1161/ATVBAHA.114.303887 24903093

[152] Barrett TJ, Murphy AJ, Goldberg IJ, Fisher EA. 2017. Diabetes-mediated myelopoiesis and the relationship to cardiovascular risk. Ann N Y Acad Sci 1402:31–42. doi:10.1111/nyas.13462 28926114 PMC5659728

[153] Edgar L, Akbar N, Braithwaite AT, Krausgruber T, Gallart-Ayala H, Bailey J, Corbin AL, Khoyratty TE, Chai JT, Alkhalil M, et al.. 2021. Hyperglycemia induces trained immunity in macrophages and their precursors and promotes atherosclerosis. Circulation 144:961–982. doi:10.1161/CIRCULATIONAHA.120.046464 34255973 PMC8448412

[154] Bekkering S, Stiekema LCA, Bernelot Moens S, Verweij SL, Novakovic B, Prange K, Versloot M, Roeters van Lennep JE, Stunnenberg H, de Winther M, Stroes ESG, Joosten LAB, Netea MG, Riksen NP. 2019. Treatment with statins does not revert trained immunity in patients with familial hypercholesterolemia. Cell Metab 30:1–2. doi:10.1016/j.cmet.2019.05.014 31204280

[155] van der Heijden CDCC, Groh L, Keating ST, Kaffa C, Noz MP, Kersten S, van Herwaarden AE, Hoischen A, Joosten LAB, Timmers HJLM, Netea MG, Riksen NP. 2020. Catecholamines induce trained immunity in monocytes in vitro and in vivo. Circ Res 127:269–283. doi:10.1161/CIRCRESAHA.119.315800 32241223

[156] Brown GD, Gordon S. 2001. Immune recognition. A new receptor for beta-glucans. Nature 413:36–37. doi:10.1038/35092620 11544516

[157] Tang C, Kamiya T, Liu Y, Kadoki M, Kakuta S, Oshima K, Hattori M, Takeshita K, Kanai T, Saijo S, Ohno N, Iwakura Y. 2015. Inhibition of dectin-1 signaling ameliorates colitis by inducing lactobacillus-mediated regulatory T cell expansion in the intestine. Cell Host Microbe 18:183–197. doi:10.1016/j.chom.2015.07.003 26269954

[158] Netea MG, van der Meer JWM. 2017. Trained immunity: an ancient way of remembering. Cell Host Microbe 21:297–300. doi:10.1016/j.chom.2017.02.003 28279335

[159] Sánchez-Ramón S, Conejero L, Netea MG, Sancho D, Palomares Ó, Subiza JL. 2018. Trained immunity-based vaccines: a new paradigm for the development of broad-spectrum anti-infectious formulations. Front Immunol 9:2936. doi:10.3389/fimmu.2018.02936 30619296 PMC6304371

[160] van Leent MMT, Meerwaldt AE, Berchouchi A, Toner YC, Burnett ME, Klein ED, Verschuur AVD, Nauta SA, Munitz J, Prévot G, et al.. 2021. A modular approach toward producing nanotherapeutics targeting the innate immune system. Sci Adv 7:10. doi:10.1126/sciadv.abe7853 PMC7935355 33674313

[161] Kaufmann E, Khan N, Tran KA, Ulndreaj A, Pernet E, Fontes G, Lupien A, Desmeules P, McIntosh F, Abow A, Moorlag S, Debisarun P, Mossman K, Banerjee A, Karo-Atar D, Sadeghi M, Mubareka S, Vinh DC, King IL, Robbins CS, Behr MA, Netea MG, Joubert P, Divangahi M. 2022. BCG vaccination provides protection against IAV but not SARS-CoV-2. Cell Rep 38:110502. doi:10.1016/j.celrep.2022.110502 35235831 PMC8858710

[162] Adams K, Weber KS, Johnson SM. 2020. Exposome and immunity training: how pathogen exposure order influences innate immune cell lineage commitment and function. Int J Mol Sci 21:8462. doi:10.3390/ijms21228462 33187101 PMC7697998

[163] Piasecka B, Duffy D, Urrutia A, Quach H, Patin E, Posseme C, Bergstedt J, Charbit B, Rouilly V, MacPherson CR, Hasan M, Albaud B, Gentien D, Fellay J, Albert ML, Quintana-Murci L, Milieu Intérieur Consortium. 2018. Distinctive roles of age, sex, and genetics in shaping transcriptional variation of human immune responses to microbial challenges. Proc Natl Acad Sci U S A 115:E488–E497. doi:10.1073/pnas.1714765115 29282317 PMC5776984

[164] Nardini C, Moreau JF, Gensous N, Ravaioli F, Garagnani P, Bacalini MG. 2018. The epigenetics of inflammaging: the contribution of age-related heterochromatin loss and locus-specific remodelling and the modulation by environmental stimuli. Semin Immunol 40:49–60. doi:10.1016/j.smim.2018.10.009 30396810

